# Coalitions and Their Negative Consequences: An Examination in Service Failure-Recovery Situations

**DOI:** 10.1177/10946705231163884

**Published:** 2023-03-30

**Authors:** Holger Roschk, Masoumeh Hosseinpour, Jan Breitsohl

**Affiliations:** 1 Aalborg University Business School, 1004 Aalborg University, Aalborg, Denmark; 2 Department of Management, 1006 Aarhus University, Aarhus, Denmark; 3 Adam Smith Business School, 3526 University of Glasgow, Scotland, UK

**Keywords:** service failure and recovery, coalitions, triads, text mining, affective tone, satisfaction

## Abstract

The social nature of customer experiences creates complex and potentially detrimental dynamics in failure situations, such as when other customers side with the complainer or the firm. The present research is the first to analyze such coalitions and their consequences. We conceptualize a triad composed of a complainer, a service employee, and one or multiple others as a third actor. A field study of consumer complaints on social media shows that coalitions occur in 32% of cases, negatively shifting the affective tone of an online conversation from approximately neutral to negative. Both third actor–complainer and third actor–service employee coalitions independently deteriorate the affective tone, their individual effects are not additive, and the third actor–complainer coalition exerts the larger impact of both coalitions. Two experiments reveal that complainers feel betrayed by the third actor when this actor sides with the service employee (vs. the complainer), which strengthens complainers’ satisfaction with taking steps as a recovery effort by the firm and weakens satisfaction with an offered apology. This research provides managerial insights into the practical significance of coalition effects, how coalitions impair firm response effectiveness, and under which conditions different responses sustain their effectiveness. It also presents several avenues for future research.

## Graphical abstract

**Graphical Abstract:**
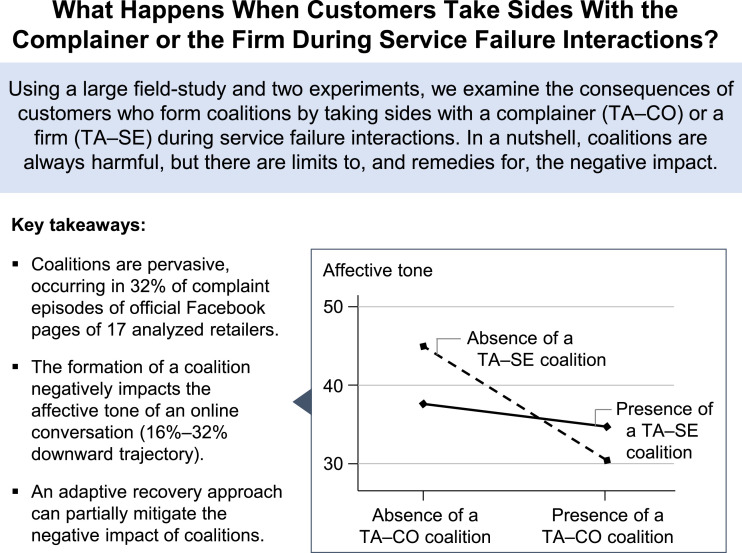

*Sarah: LEGO, I have a complaint!! I bought 3 of the Super Mario Adventure sets for our kids and there is no instruction booklet in any box. I am VERY DISAPPOINTED.*

*Eleni: Sarah, just download the instructions for them, what is your problem? We live in a paperless age, get used to it.*

*LEGO: We’re sorry to hear that you were disappointed with us. We use digital booklets because they allow us to offer additional features like zooming in. We fully understand that some of our customers prefer a physical instructions book, and we apologize for the inconvenience that you and your children have experienced.*

The above conversation, taken from LEGO’s Facebook page, illustrates how a firm that responds to a complainer is confronted with a scenario in which others take sides with the complainer or, as is the case here, with the firm—that is, they form a coalition. Coalitions remain unexplored in the service failure and recovery domain, although prior research on other individuals who act on the firm’s behalf (Weitzl and Hutzinger 2017), present a solution to the complainer (Kim and Baker 2020), or engage in hostility toward the complainer (Bacile et al. 2018) already implies the notion of coalitions. From a sociopsychological perspective, coalitions are a pervasive phenomenon with the potential to impact and disrupt social dynamics (Komorita and Chertkoff 1973). Understanding social dynamics that involve others in a service failure context is of scholarly and managerial interest (Khamitov, Grégoire, and Suri 2020), and the increasingly social nature of customer experiences creates ample opportunities for coalitions when failures occur (Lemon and Verhoef 2016). In fact, up to 53% of customers complain through social media, reaching an average of 865 connections (Customer Rage Study 2020; Social Media Today 2020). Similarly, others are often part of the experience in brick-and-mortar environments as well, for example, in shared spaces (e.g., in a theater) or via continuous social media messaging on mobile devices (e.g., Twitter postings; Grégoire and Mattila 2021).

Figure 1 illustrates how our study of coalitions differs from previous work that has examined additional individuals besides a complainer and a service employee, conceptualized here as a third actor. A key distinction is whether the third actor is studied in a triad or a triadic context, with coalitions occurring in triads. Triads (also referred to as triadic structures) represent an interlinked three-actor system. The focal criterion is that one actor connects two relations so that they influence each other (Vedel, Holma, and Havila 2016). For example, Kim and Baker (2020) found that complainers who receive help from another customer transfer the positive experience into their interaction with the service employee. The two relations are connected through the complainer, who acts as a conduit. Triadic contexts represent constellations in which a third actor has a bearing on the situation. The focal criterion is the existence of relations between individuals who are not connected; thus, influence is based on a shared context (Vedel, Holma, and Havila 2016). For example, others may observe that firms react with humor to a complaint and assess the firm based on context alone (Béal and Grégoire 2022). Although there is a contextually relevant association, it remains unknown how the relations in that situation depend on each other (i.e., which role the actors play). Therefore, triadic contexts can be viewed as the background in which triads can be formed and evolve.

**Figure 1.**
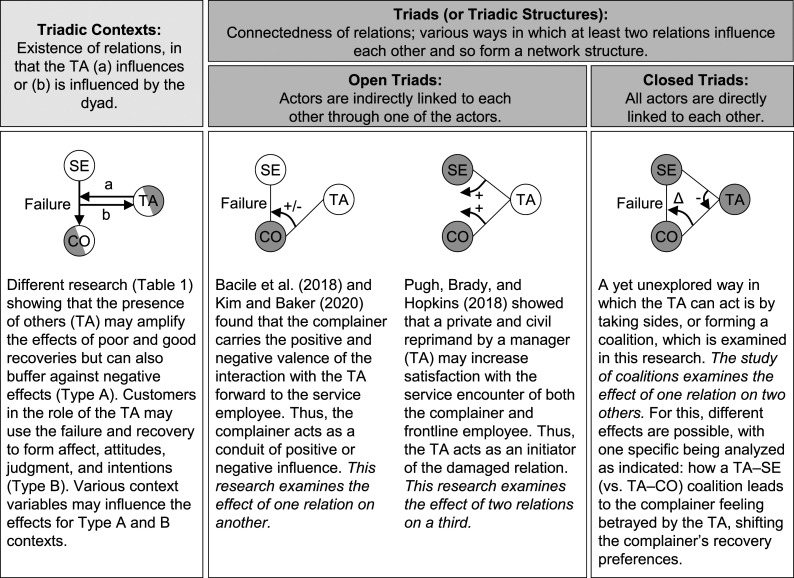
Triadic contexts, triads, and prior research. *Notes:* SE, service employee; CO, complainer; TA, third actor. The unit of analysis is marked in gray, with semicircles indicating that one of the two actors is analyzed, depending on the type of the triadic context (a: CO, b: TA). Representative sources for the conceptualization are Ritter (2000) and Vedel, Holma, and Havila (2016).

The present research is the first to examine how coalitions impact service failure and recovery settings. We conceptualize a triad as composed of a complainer, a service employee, and a third actor who comprises one or more customers. In this triad, two coalitions are conceivable: between the third actor and complainer (TA–CO coalition) and between the third actor and service employee (TA–SE coalition). We study these two coalitions and their consequences in the triad, as well as for the complainer in specific, using a field study and two experiments. Research on triads remains scarce but is needed. Previous work has predominantly focused on dyads, while some other studies have investigated triadic contexts as a background to triads; however, these have not examined triads per se, as done in the present research (Figure 1). Furthermore, there is a lack of research on social dynamics in customer experiences and, particularly, failure settings (Khamitov, Grégoire, and Suri 2020; Lemon and Verhoef 2016). Triads offer a means to capture such dynamics, as they account for interrelationships among multiple actors. Typically, research focuses on triads because the key conceptual advancement lies in extending the actors from two (dyad) to three (triad), while further extensions (e.g., three to four actors) offer comparably less (Vedel, Holma, and Havila 2016).

As a first contribution, Study 1 shows that coalitions are a pervasive phenomenon and provides insight into their effect patterns in a triad. Analyzing retailers’ official Facebook pages, we find that coalitions occur in 32% (±3.5%) of complaint episodes, shifting the affective tone of a conversation from approximately neutral to negative. While both TA–CO and TA–SE coalitions deteriorate the affective tone, their effects are not additive in that their joint effect does not exceed their individual effects. Comparing the two coalitions, TA–CO exhibits a larger negative impact, which might be weakened by TA–SE. By focusing on the triad as a unit of analysis and using behavioral field data, Study 1 offers robust insights which have been called for in service research (Grégoire and Mattila 2021; Vedel, Holma, and Havila 2016). Moreover, it expands previous work on triads by considering the triad as closed and the third actor as multiple individuals.

As a second contribution, our two experiments (Studies 2 and 3) demonstrate that, compared to the TA–CO coalition, the TA–SE coalition increases complainers’ feelings of betrayal by the third actor. Betrayal impacts complainers’ satisfaction with the recovery offered by the firm, strengthening the effect of taking steps to solve the problem and weakening that of an apology. This finding complements Study 1 results for the triad by uncovering coalition effects for the relations within the triad. Hereby, the coalition-induced change in recovery preferences indicates one way in which triad members are connected. Furthermore, our findings on the explanatory value of perceived betrayal in the triad and its novel conceptualization that focuses on the third actor suggest broadening its scope to three-actor systems and entities other than the firm. We also advance the small body of work that connects two or more relations, offering a promising angle for building knowledge on social dynamics.

Since coalitions have so far remained unexplored, our third contribution lies in offering novel insights for practitioners. First, coalitions lead to a downward trajectory of 16%–32% in the affective tone of an online conversation after a complaint. While both TA–CO and TA–SE coalitions are harmful, there also appears a lower limit in the coalition-induced downward shift. Second, the occurrence of coalitions impairs firms’ recovery efforts in online conversations and shifts complainers’ recovery preferences. Third, while findings on firm responses in triads are scarce, our results corroborate the effectiveness of accommodative response content and personal, positive response styles in the absence of a coalition. Firm responses might also mitigate some of the detrimental impact of subsequently present coalitions. The results suggest an adaptive recovery approach in line with prior research (e.g., Nazifi et al. 2021). Finally, we present an agenda for future research.

## Theoretical Development

### Triadic Structures

We conceptualize triads as a structural phenomenon and begin our discussion with the focal dyad for which four possible constellations can be distinguished in the business-to-customer domain: a customer transgressing against another customer (customer-to-customer conflicts; Brief et al. 2005) or against an employee (customer incivility; Henkel et al. 2017), and an employee transgressing against a customer (service failure and recovery; Gelbrich and Roschk 2011) or another employee (employee incivility; Porath, MacInnis, and Folkes 2011). The four constellations can be seen as prototypical instances of the focal dyad. In particular, research on service failure and recovery—the focus of this study—seeks to integrate different failures (e.g., on part of the firm, service employee, or brand) to unite existing conceptualizations of provider-driven negative events (Khamitov, Grégoire, and Suri 2020). We define the focal dyad as a service firm’s failure that impacts the complainer. Next, we discuss the transition from a dyad to a triad, the triadic nature of prior research, and other triadic conceptualizations.

Triads are based on (i) the association of three actors and (ii) the connection of at least two relations (Vedel, Holma, and Havila 2016). The association criterion describes the transition from a dyad to a triadic context. Association refers to the existence of relations in that the third actor influences or is influenced by a dyad; without association, the third actor would have no bearing on the situation (Vedel, Holma, and Havila 2016). To qualify as a triad, the existence of relations is not enough; they also need to be connected. Connectedness describes the way in which two or more relations influence each other (Ritter 2000). The connectedness of relations takes various forms (see Ritter 2000 for a systematization of 10 constellations) and can be illustrated through the different roles of the connecting actor. For example, the connecting actor may carry the experience of one relation into another (role of a conduit) or use its relation with the other two actors to bring them together (role of an initiator; see also Figure 1).^1^ Thus, a triad is defined as a structure in which at least two relations are connected among the three associated actors (Vedel, Holma, and Havila 2016).

Based on this conceptualization, prior research in the service failure and recovery domain is traditionally dyadic, as the third actor is missing. This also applies to analyses of the interaction between a group of customers, as the complaining actor, and the service firm (e.g., Albrecht et al. 2019). Table 1 provides an overview of the studies that have included the third actor. Using the defining criteria of triads, most studies can be seen as triadic in context in that they analyze how the presence of a third actor affects the complainer–service employee interaction, or is affected by it. These studies are important because they establish the existence of relations between the focal dyad and third actor. To date, triads have been rarely studied. These studies are distinct in that they look at the connectedness of relations, examining the effect of one relation on another (e.g., Kim and Baker 2020) or that of two relations on a third (Pugh, Brady, and Hopkins 2018), as illustrated in Figure 1.

**Table 1.** Overview of Prior Service Recovery Literature Examining Triadic Contexts and Structures.

Study	Triadic Setting	Triad Type	Structural Effect	Unit of Analysis	Third Actor^a^	Third Actor Studied as	Independent Variable(s)^b^	Industry Setting	Non-scenario Experimental Data? Which?^c^	Field Data for Proposed Effects?	Behavior Studied? What?
Bonifield and Cole (2008)	Triadic context (A)	—	—	CO	Present others^N^	Single unit	Presence of a downward social comparison	2 firms (restaurant, air travel)	No	No	No
Chen et al. (2020)	Triadic context (A)	—	—	CO	Present others (same problem)^V,P^	Single unit	Presence of others' complaint	2 firms (air travel, retail)	No	No	No
Chen, He, and Alden (2014)	Triadic context (A)	—	—	CO	Present others^P^	Single unit	Presence of other customers	1 firm (bookstore)	No	No	No
He et al. (2017)	Triadic context (A)	—	—	CO	Present others^P^	Single unit	Presence of other customers	1 firm (coffee shop)	No	No	No
Schaefers and Schamari (2016)	Triadic context (A)	—	—	CO	Present others^V^	Single unit	Presence of others	2 firms (car brand, telephone service)	No	No	No
Béal and Grégoire (2022)	Triadic context (B)	—	—	TA	Observer of the failure^V^	Individual	Affiliative (vs. aggressive) humor	1 blog, 1 firm (Internet service provider)	Yes, text-mining	Yes	Yes, likes and retweets
Hogreve, Bilstein, and Hoerner (2019)	Triadic context (B)	—	—	TA	Observer of the failure^V^	Individual	Transparency of service recovery	2 firms (food delivery, Internet provider)	No	No	No
Hutzinger and Weitzl (2021)	Triadic context (B)	—	—	TA	Observer of the failure^V^	Individual	Marketer (vs. customer) recovery, recovery responses	1 firm (coffee house)	No	No	No
Javornik, Filieri, and Gumann (2020)	Triadic context (B)	—	—	TA	Observer of the failure^V^	Individual	Conversational human voice (vs. corporate voice)	2 firms (air travel, car rental)	No	No	No
Johnen and Schnittka (2019)	Triadic context (B)	—	—	TA	Observer of the failure^V^	Individual	Accommodative (vs. defensive) response	20 + 1 (various industries + apparel)	Yes, text-mining	Yes	Yes, positive comments and likes
Mantovani, Korelo, and Ibarra (2018)	Triadic context (B)	—	—	TA	Observer of the failure^P^	Individual	Close (vs. distant) relationship to the victim	2 firms (telephone service, restaurant)	No	No	No
Mattila, Hanks, and Wang (2014)	Triadic context (B)	—	—	TA	Observer of the failure^P^	Individual	Good (vs. poor) service recovery	2 firms (restaurant, air travel)	No	No	No
Sharma, Jain, and Behl (2020)	Triadic context (B)	—	—	TA	Observer of the failure (distant)^V,P^	Individual	Ethical service transgressions	1 firm (air travel)	Yes, netnography^d^	No	No
Van Vaerenbergh, Vermeir, and Larivière (2013)	Triadic context (B)	—	—	TA	Observer of the failure^P^	Individual	Presence of observing a service failure and recovery	3 firms (restaurant, retail, hotel)	No	No	No
Wan and Wyer (2019)	Triadic context (B)	—	—	TA	Observer of the failure^V,P^	Individual	Incidental similarity to the service employee (vs. complainer)	4 firms (restaurant, retail, book store, hotel)	Yes, field experiment	Yes	No
Weitzl and Hutzinger (2017)	Triadic context (B)	—	—	TA	Observer of the failure^V^	Individual	Accommodative and defensive responses	1 firm (coffee house)	No	No	No
Zhao, Jiang, and Su (2020)	Triadic context (B)	—	—	TA	Observer of the failure^V^	Individual	Apologetic (vs. denial) recovery approach	3 firms (travel, retail, smartphone)	No	No	No
Bacile et al. (2018)	Triad	Open	Conduit	CO	Uncivil other customer^V^	Individual	Justice perceptions of the complainer towards the uncivil other	1 firm (restaurant)	Yes, netnography^d^	No	No
Kim and Baker (2020)	Triad	Open	Conduit	CO, TA	Civil other customer (same problem)^P^	Individual	Other customers' co-recovery (none vs. informational vs. emotional)	1 firm (hotel)	No	No	No
Pugh, Brady, and Hopkins (2018)	Triad	Open	Initiation	CO, SE	Manager^P^	Individual	Presence of a reprimand by manager	2 firms (restaurant, theater)	No	No	No
Present study	Triad	Closed	Coalition	Triad, CO	Present others^V,P^	Multiple and single individuals	Presence of coalitions	17 + 1 firms (food retailers + apparel)	Yes, text-mining	Yes	Yes, affective tone

^a^N, narratively present; P, physically present; V, virtually present.

^b^Adapted to fit the logic of the triadic context; presence refers to a presence versus absence comparison.

^c^Including scenario-based surveys by Bacile et al. (2018) and Sharma, Jain, and Behl (2020).

^d^Purpose was to introduce the phenomenon under study.

*Notes.* Triadic contexts classified according to Figure 1 in Types A and B; CO, complainer; SE, service employee; TA, third actor.

The conceptualization of triadic contexts and triads shares similarities and differences with other approaches, particularly that proposed by Siltaloppi and Vargo (2017). Similar across both approaches is the description of triadic contexts, yet their treatments differ. While the structural definition of triads delineates them from triadic contexts, Siltaloppi and Vargo (2017) did not draw such a distinction. However, to examine coalitions, this distinction is required, since coalitions occur only in triadic structures (Gamson 1961; Vedel, Holma, and Havila 2016).

### Actors and Coalitions

The actors of the triad in a failure situation comprise a complainer, a service employee, and a third actor. We define the complainer as the customer who is primarily subjected to a failure and the service employee as the firm’s representative to whom the complaint is directed. The third actor is defined as one or more other individuals, comprising socially and virtually present others (Table 1), who influence or are influenced by the complainer and service employee. This definition includes observers of the failure situation who witness other customers’ failure incidents. The term observer needs to be delineated from the term bystander. While an observer implies someone paying attention to a failure, a bystander refers to someone being merely present (Fischer et al. 2011). This means that bystanders may (e.g., when being an observer; Hutzinger and Weitzl 2021) but not necessarily need to represent a third actor (e.g., when an association does not exist).

A coalition is a process in which two actors (humans or firms) form a temporary alliance (e.g., joint action, agreement to cooperate, and endowment of another actor) with the aim of exerting influence within a given setting (Gamson 1961; Komorita and Chertkoff 1973; Thibaut and Kelley 1959; Vedel, Holma, and Havila 2016). Applied to the context of this study, we define a coalition as a process in which another customer takes sides with either the service employee or the complainer during a service failure incident to influence the situation. This line of thought treats coalitions as a phenomenon within a triad. Research has also referred to coalitions as a type of triad, specifying the triad as acting like one entity (Vedel, Holma, and Havila 2016). Considering the entire triad as a coalition includes all triad actors instead of just two. In what follows, we focus on coalitions between two actors as the smallest possible coalition in a triad, which is in line with other research on coalitions (Gamson 1961).

### Hypotheses

#### Overview

We discuss the expected negative impact of coalitions (H_1_ and H_2_) in the triad and how coalitions interfere with the effectiveness of firms’ responses to complaints (H_3_). We then focus on the complainer and explain how coalitions can impact the complainer’s satisfaction with the recovery offered by the firm (H_4_).

#### Coalitions’ impact on the triad

**Figure 2.**
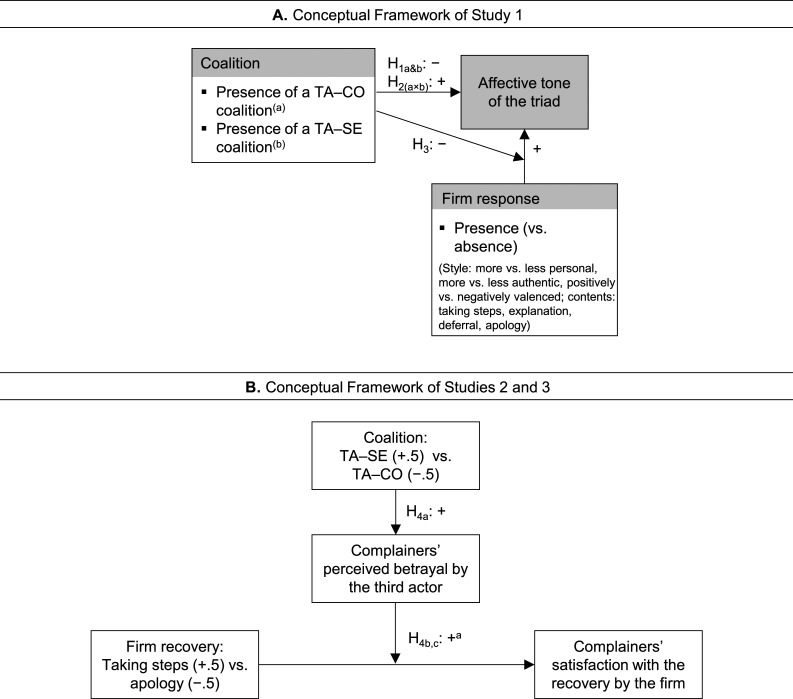
Overview of studies and hypotheses. *Notes*: ^a^The expected sign of the interaction term, indicating that higher values of complainers’ perceived betrayal by the third actor favors the effect of taking steps on complainers’ satisfaction with the recovery by the firm and disfavors that of an apology.

Figure 2, panel A, shows the conceptual framework for the effects of coalitions on the valence of the affective tone of the triad within an online context. We focus on affective tone as a salient, feeling-based expression of the social dynamics in a group (Zaccaro, Rittman, and Marks 2001). Moreover, negative emotions are commonly observed among complainers, strongly related to their reactions, and a key factor in failure episodes that go viral (Herhausen et al. 2019; Valentini, Orsingher, and Polyakova 2020).

Realistic group conflict theory is widely used to explain how group behavior is shaped by conflicts (Sherif et al. 1951), and recent research supports its applicability in online environments (Brief et al. 2005; Neubaum and Krämer 2017). The theory suggests that tension is created when groups, due to a conflict, experience an opinion imbalance. Such tension leads to the group as a whole experiencing negative attitudinal, behavioral, and emotional consequences (Jackson 1993). This is because group members are forced to evaluate their own position as a consequence of an underlying fear that they will become part of an undesired minority group (Vargas-Salfate et al. 2018). In line, research has shown that opinion imbalances in online discussions create tensions, and that a homogenous opinion climate reflects the preference for a state of harmony (Neubaum and Krämer 2017). In the present context, a voiced service failure can be seen as a conflict between a complainer and a service employee. When a third actor joins in and takes sides, an opinion imbalance results. This can then lead to tension and force the triad members to consider their own standpoint on the failure, which likely increases the expression of negative attitudes and emotions.^2^ Thus,

**H_1_:** The presence (vs. absence) of (a) a TA–CO and (b) a TA–SE coalition leads to a more negative affective tone of the triad.

When the third actor comprises multiple individuals, as is the case on social media, multiple coalitions may occur in a single complaint episode. Given the opposing nature of the TA–CO and TA–SE coalitions, an important question relates to how both coalitions together affect the triad. Realistic group conflict theory suggests that as the number of conflicts increases, the negative consequences for a group as a whole also increase (Yang, van de Vliert, and Jehn 2018). Thus, the joint occurrence of the TA–CO and TA–SE coalitions can intensify the conflict and lead to a more negative affective tone than that of each coalition. Yet, the extent to which the individual effects of both coalitions are additive remains unclear.

Large communities on social media have a strong likelihood of an excessive number of competing standpoints (Walther 2018). Such complex interactions can exceed the cognitive resources of group members and cause discomfort (Fu et al. 2020). In response, group members may engage in selective processing; that is, they deliberately ignore information that surpasses their cognitive resources and creates discomfort (Arceneaux, Johnson, and Murphy 2012). Recent findings have indicated that such behavior can occur on social media (Jeong et al. 2019). In the present context, after being exposed to a coalition, the triad members may process further coalitions only selectively to balance their cognitive effort. In particular, additional coalitions that express competing standpoints may be ignored to avoid discomfort. As a result, the combined effect (or presence) of the TA–CO and TA–SE coalitions on affective tone will be less negative than the sum of their individual effects. Statistically, this represents a positive interaction between the presence of a TA–CO coalition and that of a TA–SE coalition, assuming a negative impact of both coalitions individually on affective tone. Thus,

**H**_**2**_**:** The joint presence of a TA–CO and a TA–SE coalition leads to a less negative affective tone of the triad than the sum of both coalitions individually.

From a managerial viewpoint, it is of interest how coalitions interfere with the effectiveness of firms’ responses to complaints. Personal response styles and accommodative response content are favorable recovery strategies in online contexts (Abney et al. 2017; Johnen and Schnittka 2019). Therefore, the presence (vs. absence) of a firm response may yield a more positive affective tone. In addition, we propose that the presence of a coalition weakens the effectiveness of a firm response. Similar to our previous reasoning, the formation of either coalition, TA–CO or TA–SE, is likely to increase the complexity of the interaction process (Jeong et al. 2019). As a result, triad members may selectively process the conversation and focus more on negative stimuli, such as coalitions, which also outweigh positive stimuli, such as a firm’s response (negativity bias; Rozin and Royzman 2001). Accordingly, a firm’s response may lose its effectiveness due to the presence of a coalition. Thus,

**H**_**3**_**:** The presence (vs. absence) of a coalition weakens the effectiveness of firm responses to positively influence the affective tone of the triad.

#### Coalitions’ impact on the complainer

So far, we have theorized the impact of coalitions on the triad as a whole. Next, we discuss how coalitions affect the relations within the triad. We explain the analyzed variables, their conceptual relations, and the expected effects.

Since coalitions can influence the relations in various ways (Ritter 2000; Thibaut and Kelley 1959), we anchor the coalition effects on the complainer as the actor most directly affected by the failure. We focus on transaction-specific satisfaction, defined as the complainer’s evaluation of a firm’s failure response (Albrecht et al. 2019; Pugh, Brady, and Hopkins 2018). It aligns with the affective tone from the previous hypotheses by being feeling-based, while also including cognitive elements that are relevant in service failure settings (Khamitov, Grégoire, and Suri 2020). As recovery efforts, we examine taking steps (communication that the firm will try to solve the problem) and apology (an empathic expression of remorse for a failure; Roschk and Gelbrich 2014).^3^ Taking steps and an apology address different coping strategies of the complainer, being either problem- or emotion-oriented respectively (Duhachek 2005).

Figure 2, panel B, depicts our conceptual framework, illustrating how the recovery–satisfaction link is moderated by whether a coalition is present, and the moderating effect is explained by perceived betrayal. The variable constellation represents a mediated moderation, which is concerned with the process (i.e., perceived betrayal) that is responsible for the moderation (i.e., the effect of coalition; Baron and Kenny 1986; Muller et al. 2005). Mediated moderation, particularly treating perceived betrayal as a moderator, provides an appropriate conceptualization to account for the triadic structure underlying these effects. From the complainer’s perspective, perceived betrayal relates to *the actions of the third actor* who accompanies the complainer, while the complainer’s satisfaction is expressed toward *the firm’s actions* (i.e., the recovery offered by the firm). Perceived betrayal is typically researched as a reaction to something that the service provider did (Khamitov, Grégoire, and Suri 2020). Thus, employing perceived betrayal as a reaction to something the third actor did presents a novel contextual variable to the recovery–satisfaction link that extends current knowledge.

Based on the conceptual framework presented in Figure 2, panel B, we propose that a TA–SE (vs. TA–CO) coalition increases complainers’ perceived betrayal by the third actor. Perceived betrayal describes the belief that someone has intentionally violated a relationship norm (Grégoire and Fisher 2008). Relativistic group theory suggests that individuals hold a normative expectation of getting support from fellow group members (Benard 2012). For the complainer–third actor relation, the complainer may perceive such a norm breach when the third actor sides with the other party (service employee) rather than with the complainer. Thus,

**H**_**4a**_**:** A TA–SE (vs. TA–CO) coalition increases complainers’ perceived betrayal by the third actor.

Furthermore, we propose that a complainer’s perceived betrayal by the third actor moderates the complainer’s expressed satisfaction with the firm’s recovery. Realistic group conflict theory suggests that when group members fail to support an individual’s opinion, a homogenous opinion climate becomes less conceivable and thus incurs emotional stress for the individual. The individual then seeks a problem-focused coping strategy since it allows to move beyond the emotional processing of the conflict and leave it behind (Benard and Doan 2011). In contrast, an emotion-focused coping strategy prolongs the emotional processing, making it harder for the individual to move on (Levine, Moreland, and Ryan 1998). In our case, the complainer experiences emotional stress when betrayed by the third actor. To the complainer, a problem-focused recovery by the firm (taking steps to address the problem) is then likely to be more satisfying, while an emotion-focused recovery (an empathic apology) is less satisfying; the former shifts the complainer’s focus away from the emotional processing of the conflict, whereas the latter prolongs, if not intensifies, the processing. Thus,

**H**_**4b,c**_**:** Complainers’ perceived betrayal by the third actor (b) strengthens taking steps’ positive effect on satisfaction and (c) weakens apology’s positive effect.

H_4a_ and H_4b,c_ theorize the chain of effects of a coalition, through perceived betrayal, on the recovery–satisfaction link. To establish mediated moderation, we follow its core idea of explaining a focal moderation, which in our case is that of a coalition (Muller, Judd, and Yzerbyt 2005). Therefore, we also expect an interaction between coalition and recovery to follow a similar pattern, so that a TA–SE (vs. TA–CO) coalition strengthens the recovery effect of taking steps and weakens that of an apology. When the perceived betrayal × recovery interaction is accounted for, mediated moderation requires the coalition × recovery interaction to become less or not significant (Baron and Kenny 1986; Muller, Judd, and Yzerbyt 2005).

### Overview of Studies

We conducted one field study and two experiments. Study 1 was a text-mining study on social media, which had three objectives: (1) to gauge the frequency with which coalitions occur in real-life settings, (2) to test the impact of both coalitions on the valence of the affective tone of the triad (H_1_ and H_2_), and (3) to assess how coalitions interfere with firm response effectiveness (H_3_). Study 2 was an experiment to test if the presence of a TA–SE coalition compared to a TA–CO coalition leads to the complainer feeling betrayed by the third actor, which in turn yields a shift in the complainer’s recovery preferences (H_4_). Study 3 was a replication study for H_4_.

## Study 1

### Method

#### Database development

We scraped user comments from Facebook based on reports indicating its status as customers’ preferred choice for complaining about companies (Social Media Today 2020). We collected data from the official Facebook page of 17 international retailers, including coffee chains (e.g., Costa Coffee), fast-food joints (e.g., McDonalds), groceries (e.g., Tesco), and general retailers (e.g., Marks & Spencer; Table 2). An initial screening suggested these retailers because of their high frequency of consumer activity and retailers’ content contribution. Using Python and the scraping modules Selenium and Beautiful Soup, we generated an initial dataset of all comments made on the retailers’ Facebook pages in the United Kingdom between December 2020 and March 2021, totaling 17,191 episodes (i.e., Facebook’s predefined comment chains) and 25,374 individual comments. We then combined automated classifiers (i.e., machine-learning classification) and manual coding to identify episodes that contained a complaint. Machine-learning classification allows for the handling of considerable data, and subsequent manual coding uses human reasoning to ensure data accuracy.

**Table 2.** Definition, Data Source, and Statistical Properties of Study Variables.

Level: Variable		Definition	Measure-ment	Mean (SD)
Affective tone				
1:	Affective tone^a^	Text-based valence of affective sentiment, indicating a positive (>50) or negative (<50) emotional tone. (1–100)	LIWC dictionary	39.81 (34.98)
Number and presence of coalitions				
2:	Number of TA–CO coalitions	Number of third actors in a complaint episode that took sides with the complainer. (Number)	Manual coding	0.66 (1.22)
2:	Number of TA–SE coalitions	Number of third actors in a complaint episode that took sides with the service employee. (Number)	Manual coding	1.34 (2.78)
1:	Presence of a TA–CO coalition	Did the comment follow or institute a TA–CO coalition (i.e., presence) or was a TA–CO coalition absent at the point in the discussion. (0 = absence, 1 = presence)	Manual coding	.41 (.49)
1:	Presence of a TA–SE coalition	Did the comment follow or institute a TA–SE coalition (i.e., presence) or was a TA–SE coalition absent at the point in the discussion. (0 = absence, 1 = presence)	Manual coding	.57 (.50)
Firm response				
1:	Presence of a firm response	Whether the comment was made after (i.e., presence) a firm response or a response was not (yet) made. (0 = absence, 1 = presence)	Extraction	.22 (.41)
–^b^	More personal firm response	The firm responded in a more (e.g., addressing the complainer by name) or less personal manner. (0 = less, 1 = more personal)	Manual coding	.67 (.47)
–^b^	More authentic firm response	Text-based authenticity of the firm response, being above or below the average across all firm responses. (0 = less, 1 = more authentic)	LIWC dictionary	.36 (.48)
–^b^	Positive firm response	Text-based emotional tone of the firm response, being either positive (≥50) or negative (<50). (0 = negative, 1 = positive).	LIWC dictionary	.73 (.44)
–^b^	Taking steps	The firm has expressed that it has taken or will take specific steps to solve the problem (e.g., approached manager). (0 = no, 1 = yes)	Manual coding	.34 (.47)
–^b^	Explanation	The firm gave an explanation for the problem. (0 = no, 1 = yes)	Manual coding	.27 (.44)
–^b^	Deferral	The firm asked the complainer to contact them privately (e.g., direct message) or do so themselves. (0 = no, 1 = yes)	Manual coding	.46 (.50)
–^b^	Apology	The firm expressed remorse for what has happened. (0 = no, 1 = yes)	Manual coding	.67 (.47)
Control variables				
2:	Failure controllability	The firm could have taken steps to prevent the failure. (1 = not at all, and 7 = very much)	Manual coding	5.87 (.66)
2:	Failure stability	The cause of the failure is likely to vary or be stable over time. (1 = not at all, and 7 = very much)	Manual coding	3.50 (1.41)
2:	Outcome failure	Whether the results (outcome) or the way (process) of product or service delivery was flawed. (0 = process, 1 = outcome failure)	Manual coding	.62 (.49)
2:	Failure reversibility	To what extent the failure can be repaired or reversed (or is nonrepairable). (1 = not at all, and 7 = very much)	Manual coding	3.53 (1.56)
2:	Failure severity	The size of loss that is entailed in a failure, rendering the failure a minor or major problem. (1 = not at all, and 7 = severe)	Manual coding	4.92 (0.89)
2:	Mediators	Number of users who acknowledged the complaint and attempted at suggesting ways of fixing the problem. (Number)	Manual coding	.07 (.31)
2:	Branch: coffee chains^c^	Coffee chains, including Costa coffee and Greggs. (0 = baseline, 1 = coffee chains)	Extraction	.15 (.36)
2:	Branch: Fast-food joints^c^	Fast-food joints, including McDonalds, Burger King, Subway, and KFC. (0 = baseline, 1 = fast-food)	Extraction	.10 (.30)
2:	Branch: General retailers^c^	General and apparel retailers, including Marks and Spencer, Primark, Dr Martens, Zara, and Next. (0 = baseline, 1 = general retailers)	Extraction	.09 (.29)
2:	Brand buzz^d^	YouGov’s Brand Buzz score, measuring whether consumers heard positive (>0) or negative things (<0) about a brand. (-100–100)	YouGov	8.69 (3.68)
1:	Word count	The text length of the comment. (Number of words)	Dictionary	29.01 (29.69)
1:	Words > six letters	Percentage of words in the comment that are longer than six letters. (0–100%)	Dictionary	14.48 (10.55)
2:	Daytime	Whether the episode started during daytime or late evening hours (6:00–24:00) or nighttime (0:01–5:59). (0 = nighttime, 1 = daytime)	Extraction	.95 (.22)

^a^16 values were missing (e.g., a comment that did not contain any text) and replaced with the mean.

^b^The coding was based on the 123 episodes in which a retailer responded to the complaint. When there were multiple firm comments within an episode (e.g., conversation between firm and complainer), we combined the firm comments.

^c^Dummy variables for four categories (coffee chains, fast-food joints, general retailers, and grocery retailers). Grocery retailers (Asda, Tesco, Aldi, Sainsbury’s, Lidl, Morrisons) were the largest category (*N* = 213) and used as the baseline comparator.

^d^The score is based on the United Kingdom market and data from March 2021, thus aligning with our Facebook data.

For machine-learning classification, we used a Naive Bayes algorithm to program our classifier (Kowsari et al. 2019). After programming, the classifier had to be calibrated (i.e., trained), which required data on social media posts categorized into complaints or not. We used the data provided by Preotiuc-Pietro, Gaman, and Aletras’s (2019), which contains Twitter posts manually annotated for representing complaints or not. Although the database does not refer to Facebook posts, it provides numerous social media comments (*N* = 1,971) across different industries (e.g., retail and apparel), making it a suitable choice for training our classifier. For subsequent manual coding, we defined a complaint as an expression of dissatisfaction with a product or service performance that falls below a customer’s expectations (Khamitov, Grégoire, and Suri 2020). For training purposes, two independent judges (one research assistant and one of the authors) coded the first 35 episodes, which were identified by the automated classifier, and agreed on 88% of the cases. Disagreements were resolved through discussion.

Applied to the collected Facebook data, the machine-learning classifier identified 1,463 episodes as potential complaints in the first step. These episodes served as a pre-selection and were manually coded in the second step to identify actual complaints (separated from false automated detections). After manual coding and a discussion of disagreements, we identified 681 complaint episodes. A further data-handling step was necessary. Of the 681 complaint episodes, 358 were single complaint posts that did not receive any response. Since we were interested in the interactions following a complaint, these episodes were excluded. Consequently, the final dataset contained 323 episodes, including 1,925 comments.

#### Variables

Table 2 shows the definitions, measurement modes, and statistical properties of Study 1 variables. We again used manual coding and automated analyses. For the manually coded variables, two research assistants coded the data following the definitions in Table 2. Their agreement rate ranged from .81 to .99. Coding reliability based on Krippendorf’s alpha was .81 or larger, exceeding the .80 threshold. Coding inconsistencies of categorical variables were resolved through discussion with one of the study authors and those of continuous variables by calculating the average of the coded values. Study 1 comprised four sets of variables.

First, we captured the valence of the affective tone expressed in a comment. Text-based affective sentiments represent naturalistic expressions of positive and negative evaluations of a service experience (Berger et al. 2020). We measured affective tone through the bipolar emotional tone dimension of the LIWC-22 dictionary (LIWC; Pennebaker Conglomerates, Inc. n.d.), an established text analysis tool within social media research (Boyd et al. 2022). The emotional tone dimension was defined as the difference between positive and negative emotional dimensions and had a value ranging from 0 to 100. Values above (below) 50 indicated a more positive (negative) tone, with 50 representing a neutral tone (Boyd et al. 2022).

Second, the coalition dynamics were based on the following coding procedure. Each comment by a user was manually coded if it represented a coalition (i.e., an expression of taking side) with the complainer (TA–CO coalition) or the retailer (including the service employee; TA–SE coalition). To avoid double counts, each user within an episode could form a coalition only once. The variables, number of TA–CO coalitions and number of TA–SE coalitions, were given by the sum of the respective coalitions in an episode. The variables, presence of a TA–CO coalition and presence of a TA–SE coalition, were given by defining the coalition comment and all subsequent comments in an episode as 1 (presence) and the other comments as 0 (absence).

Third, we measured the presence of a firm response analogous to the presence of the coalitions. We further took an exploratory approach, capturing major differences in the response style and content. One difference in style was whether the firm responded in a more (vs. less) personal manner. Compared to generic response statements, personalized responses directly address the receiver as an individual, rendering a response more relatable and valuable for the complainer (Abney et al. 2017; Roschk and Gelbrich 2017). Moreover, we captured the authenticity of the firm’s response. Authenticity refers to the perceived genuineness of a message (Boyd et al. 2022), which likely enhances the credibility of a firm’s response. Based on the idea that a more positive (vs. negative) tone may guard against prolonging the negative failure event, we also assessed the affective tone of the firm’s response itself. Finally, we captured the response content by considering four strategies: taking steps, explanation, deferral, and apology. Table 2 provides the operationalization of the different response styles and contents.

Fourth, we included control variables to account for potential confounding factors that may occur in a field setting (Berger et al. 2020). The first set, failure characteristics, included causal attributions about the failure (controllability and stability), its type (outcome vs. process, reversible vs. irreversible), and its severity (Hess, Ganesan, and Klein 2003; Roschk and Gelbrich 2014). It further comprised the presence of mediators to account for other customers’ attempts to fix the complaint (Kim and Baker 2020). Further, industry characteristics included the branch and brand buzz of the retailer to account for potential differences in customer attitudes toward the retailers. Finally, language metrics contained word count and the percentage of words with more than six letters (Javornik, Filieri, and Gumann 2020). We also included daytime to capture mood variations across daytime and nighttime.

### Analysis

For testing hypotheses H_1_–H_3_, the extraction of user comments within a complaint episode yielded a nested data structure, for which hierarchical linear models (HLMs) are the preferred method. Before running the HLMs, we checked three data properties. First, the amount of variance in the affective tone that resulted from complaint episode membership was 9.0% (*p* < .001), formally requiring a hierarchical approach (Huang 2018). Second, to ensure model robustness, the major restriction is often the higher-level sample size, for which our data with 323 episodes exceeded the recommended threshold of 50 (Maas and Hox 2005). Moreover, the sample size of each level offered sufficient power (≥80%) to detect small effects (*r* = .08) of the coalition and firm response variables (Arend and Schäfer 2019). Third, we checked for multicollinearity as another threat to model robustness. Because there is no direct diagnostic in an HLM, we regressed the affective tone on the presence of a TA–CO coalition, the presence of a TA–SE coalition, the presence of a firm response, and the control variables in a conventional model, yielding a maximum variance inflation factor of 1.99 (Roschk and Hosseinpour 2020).

We specified two-level HLMs, distinguishing between variables measured at the comment level (Level 1) and episode level (Level 2; Table 2). We estimated three models. To test H_1_ and H_2_, we regressed the affective tone on the presence of a TA–CO coalition, the presence of a TA–SE coalition, their interaction (formed through multiplication), and the control variables (Model 1). Since the coalition variables were dummy coded, the main effects denoted the effect of each coalition in the absence of the other coalition, allowing us to assess the coalition effects (H_1_) independent of their interaction (H_2_) (Cohen et al. 2003). Because the affective tone of the initial complaint comment and the firm responses might differ from the remaining comments, we included two dummy variables to control for these potential confounds. To test H_3_, we added in the first step the variable presence of a firm response to the model estimations (Model 2). In the second step, we distinguished the presence of a firm response based on whether a coalition was present or absent (Model 3) and tested whether its effect differed across the coalition conditions, assessing H_3_. Theme 1 in the Web Appendix provides the formulaic representations of the models and further details.

### Results

#### Occurrence of coalitions

Across the 1,925 comments, we observed 646 instances in which a user formed a coalition with the complainer (213 coalitions) or the service employee (433 coalitions). Next, we estimated the frequency at which a complaint episode contained a coalition. Across the 323 complaint episodes, at least one coalition occurred in 218 episodes. As a comparator, we used the total number of complaints from the Facebook data (*N* = 681); this included the 323 analyzed complaint episodes and the 358 complaints, which were single posts and thus without any coalition. The frequency was 32.0% (=218/681) and the error margin was ± 3.5% (at 95% confidence), yielding a population estimate between 28.5% and 35.5% for the complaints made on the retailers’ Facebook pages.

#### Impact of coalitions

**Figure 3.**
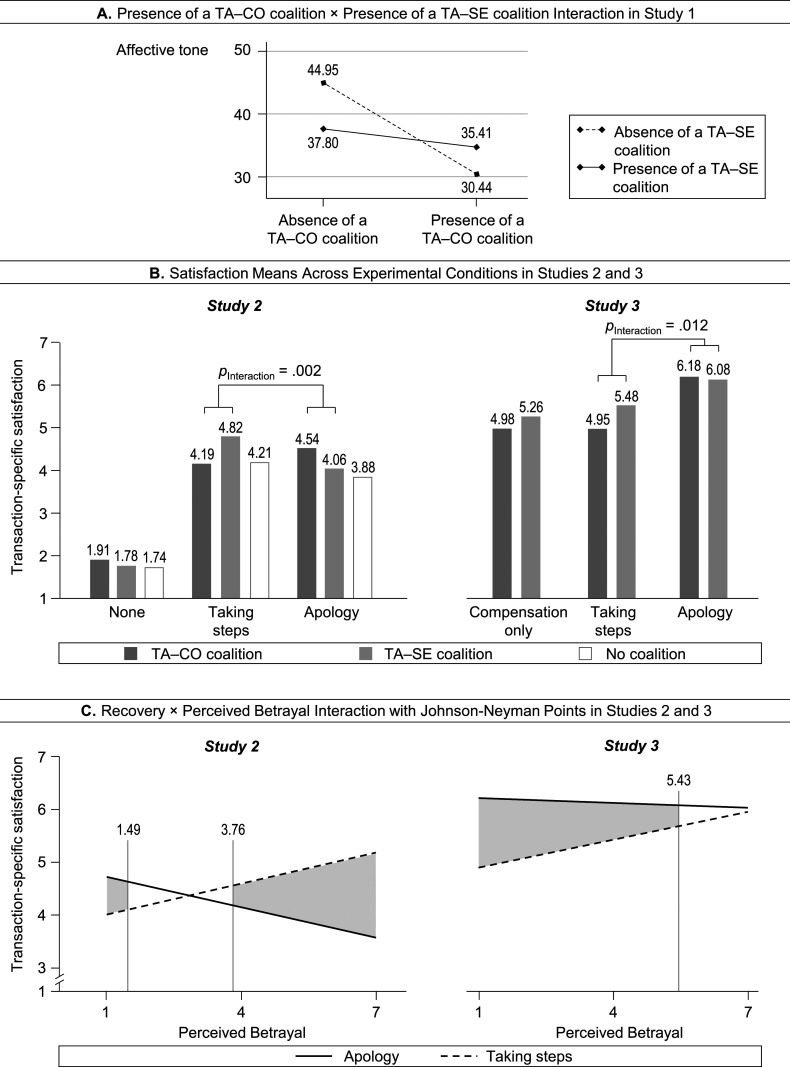
Results of Studies 1, 2, and 3. *Notes:* Means are adjusted for the control variables in all panels. In panel C, the interactions are plotted using a “floodlight” analysis of the perceived betrayal × recovery interaction.

Table 3 presents the HLM results. In Model 1, the presence of a TA–CO coalition (β = −14.51, *p* < .001) and a TA–SE coalition (β = −7.16, *p* = .034) led to a more negative affective tone, supporting H_1a_ and H_1b_. The effect of a TA–CO coalition was larger than that of a TA–SE coalition (*p*_Δcoeff_^4^ = .007). The results also indicated a positive interaction between both coalitions (β = 12.12, *p* = .002). As shown in Figure 3, panel A, the presence of a TA–CO coalition shifted the affective tone from 44.95 to 30.44 (*d* = −.41) and that of a TA–SE coalition from 44.95 to 37.80 (*d* = −.20), both in the absence of the respective other coalition. As expected, the presence of both coalitions yielded an affective tone of 35.41 (*d* = −.27), which is similar to each coalition alone, supporting H_2_. In addition, the affective tone tended to be less negative in the presence of both coalitions compared to a TA–CO coalition alone (35.41 vs 30.44, respectively; *p* = .071), indicating a partial buffering effect of a TA–SE coalition.

**Table 3.** HLM Results of the Impact of Coalitions and Firm Responses on Affective Tone.

Dv: Affective Tone	Expected Direction	1: Presence of Coalitions			2: Presence of a Firm Response			3: Presence of a Firm Response Before and After the First Coalition		
		Coeff.	SE	*P*	Coeff.	SE	*p*	Coeff.	SE	*p*
Intercept (level 2)		59.00	13.04	<.001	57.20	13.09	<.001	55.57	12.89	<.001
Coalitions										
Presence of a TA-CO coalition	H_1a_: −	−14.51	3.32	<.001	−14.17	3.26	<.001	−11.01	4.00	.006
Presence of a TA-SE coalition	H_1b_: −	−7.16	3.38	.034	−6.51	3.34	.052	−3.57	4.01	.373
Presence of a TA-CO coalition × presence of a TA-SE coalition	H_2_: +	12.12	3.95	.002	11.46	3.93	.004	8.64	4.52	.056
Firm response										
Presence of a firm response		—	—	—	2.92	2.44	.231	—	—	—
Presence of a firm response under …										
… (a) absence of a coalition^a^	H_3_: (a) > (b)	—	—	—	—	—	—	8.46	4.81	.079
… (b) presence of a coalition^a^		—	—	—	—	—	—	.32	2.47	.896
Control variables^b^										
Failure controllability		−.79	1.66	.635	−.54	1.71	.753	−.68	1.68	.685
Failure stability		1.23	.72	.087	1.29	.72	.076	1.31	.72	.068
Outcome failure		.51	2.20	.815	.52	2.19	.811	.50	2.18	.819
Failure reversibility		.13	.82	.876	.12	.82	.880	.13	.81	.872
Failure severity		−1.07	1.55	.490	−1.20	1.54	.437	−1.19	1.55	.442
Mediators		3.86	2.39	.107	3.71	2.40	.124	4.34	2.46	.078
Branch: coffee chains		−2.85	3.19	.373	−2.79	3.18	.380	−2.98	3.14	.344
Branch: Fast-food		.04	4.05	.993	.39	4.03	.923	−.01	4.04	.998
Branch: General retailers		−.10	4.53	.983	.37	4.57	.935	.31	4.60	.946
Brand buzz		−.15	.37	.695	−.18	.38	.638	−.20	.38	.596
Word count		−.03	.02	.160	−.03	.02	.147	−.03	.02	.151
Words > six letters		−.21	.07	.003	−.21	.07	.002	−.21	.07	.002
Daytime		−4.12	4.96	.407	−4.22	4.94	.393	−4.27	4.90	.384
Complaint comment		−5.68	3.44	.099	−4.52	3.47	.193	−1.89	4.04	.639
Firm comment		29.10	3.35	<.001	29.91	3.34	<.001	31.38	3.48	<.001
Model information										
∆Deviance (df)		167.8 (18)			169.2 (19)			171.7 (20)		
*p* of ∆deviance		<.001			<.001			<.001		
R^2c^		9.5%			9.5%			9.7%		

^a^We obtained similar results for the absence and presence of a TA–CO coalition (absence: β = 4.98, *p* = .168; presence: β = 0.64, *p* = .823) and a TA–SE coalition (absence: β = 4.98, *p* = .163; presence: β = 1.02, *p* = .717). Therefore, we did not distinguish between the two coalitions in this analysis, yielding stronger power.

^b^17 episodes comprised next to the focal problem a reference to other customers. We checked via a dummy variable, coding these episodes as 1 (and otherwise as 0), if they influenced the results, which they did not (Models 1/2/3: γ = −2.21/−2.43/−2.25, *p* = .552/.503/.540).

^c^Calculated as reduction in total variance of the model to the null model (Huang 2018).

#### Impact of retailers’ responses

The results of Model 2 showed that the presence of a firm response did not influence affective tone (β = 2.92, *p* = .231). Yet, conditional on the absence versus presence of a coalition (Model 3), the results indicated that the presence of a firm response led to a more positive affective tone in the absence (β = 8.46, *p* = .079), but not presence, of a coalition (β = 0.32, *p* = .896), with the coefficients differing from each other (*p*_Δcoeff_ = .057). The positive effect in the absence condition requires some caution, given the relatively high *p*-value.

To gain further insights, we explored the potential negative impact of a coalition on firm response effectiveness across different response styles and contents. First, we checked which response styles (less personal, less authentic, and negative) and content (deferral) did not exert a positive effect on affective tone in the presence or absence of a coalition (*p*-values >.2). Next, we tested the impact of a coalition on the remaining responses using Model 3 as a template for each test. The presence of a coalition impaired the positive effect of more personal responses (coalition absence: β = 12.28, *p* = .024; coalition presence: β = 3.31, *p* = .275; *p*_Δcoeff_ = .053), positive responses (coalition absence: β = 10.46, *p* = .045; coalition presence: β = 0.58, *p* = .817; *p*_Δcoeff_ = .039), and responses offering to take steps, an explanation, and/or an apology (coalition absence: β = 9.43, *p* = .050; coalition presence: β = 0.47, *p* = .853; *p*_Δcoeff_ = .042). We found a similar pattern for more authentic responses (coalition absence: β = 11.35, *p* = .099; coalition presence: β = 0.75, *p* = .823; *p*_Δcoeff_ = .102). The results largely support H_3_.

Comparing the results across the models, another interesting finding emerged. While the coalition effects remained robust across Models 1 and 2, they were somewhat reduced in Model 3. This tentatively indicates that a firm’s response in the absence of a coalition mitigates the subsequent impact of coalitions.

### Discussion of Study 1

Study 1 was a text-mining study of the official Facebook pages of 17 retailers, where we observed actual behaviors in a naturalistic field setting. Our results showed that a coalition occurred in 32.0% of the complaint episodes, substantiating the notion of coalition formation as frequent dynamic in three-actor failure settings. Consistent with our hypotheses, we found that the presence (vs. absence) of both coalitions negatively impacted the affective tone of the online conversation, yielding a negative shift from an approximately neutral toward a negative tone (H_1a_ and H_1b_). The effects of both coalitions were not additive (H_2_), suggesting a lower limit to the coalition-induced downward trajectory. The presence (vs. absence) of a coalition impaired firm response effectiveness (H_3_). In addition, our results showed a larger impact of a TA–CO than a TA–SE coalition and a partial buffering effect of a TA–SE coalition; moreover, they indicated that a firm response might mitigate some of the detrimental impact of subsequently present coalitions. Study 1 also identified personal and positive response styles and accommodative response content (i.e., taking steps, explanation, and/or apology) as favorable firm responses.

To gain further insights, we next examined the coalition effects for the relations in the triad, specifically how coalitions impact complainers’ appraisal of the actions by the third actor and how this influences complainers’ satisfaction with the recovery offered by the firm (H_4_). To ensure equivalence in the settings, we used similar contexts in Study 1 and Study 2.

## Study 2

### Method

#### Experimental design

This study used a 3 (coalition: TA–CO coalition, TA–SE coalition, no coalition) × 3 (recovery: no recovery, taking steps, apology) between-subjects design. The no-recovery condition served as a control condition to rule out differences in satisfaction by the TA–CO and TA–SE coalitions in the absence of a recovery; similar was the case for the no-coalition condition and taking steps and apology. The scenarios for Study 2 were based on actual Facebook episodes captured in Study 1. We chose Tesco, one of the largest grocery retailers in the United Kingdom, as the service firm for our scenarios. Grocery retailing is an underrepresented industry that needs further research (Khamitov, Grégoire, and Suri 2020).

Using photographic illustrations, we simulated interactions on a Facebook page. The participants were asked to put themselves in the shoes of a customer who complained about his daughter getting sick after eating from one of Tesco’s Butternut Squash baby food jars. We manipulated the coalition formation by another user replying, “I would feel the same mate. Tesco needs to be more transparent, this sounds really serious” (TA–CO coalition) or “Move on, your child might be sick from something else. I buy Tesco’s baby food all the time, never had a bad experience” (TA–SE coalition). In the no-coalition condition, the other user made an irrelevant post. The participants then imagined checking the Facebook page again a little later, moving on to the recovery scenarios in which they saw one of the following: a Tesco employee who replied by advising not to use the baby food jars and offering to test them (taking steps), apologizing for what has happened and expressing empathy (apology). In the no-recovery condition, they saw three unrelated user comments. Details can be found in Theme 2 in the Web Appendix.

#### Data collection

A power analysis (G*Power; 80% power, α = .05) with a small-to-medium effect size (*d* = .35) suggested a cell size of 65 for an interaction between coalition (TA–CO, TA–SE) and recovery (taking steps, apology). Data were gathered from a crowdsource panel (Clickworker) in November 2021. The subjects included United Kingdom residents and were paid 1 pound sterling each for participation. Upon completion, we excluded 28 of the 560 respondents because they had provided suspicious answers (failed attention checks, responded at extreme speed, and gave uniform answers). The final sample included 532 participants (mean age 37.9 years, 59.8% females), who were randomly assigned to the experimental conditions. The cell sizes were adequate, ranging from 55 to 63.

#### Measures

We measured the complainers’ transaction-specific satisfaction (e.g., “Judging this particular service encounter, I am satisfied”; α = .97) and perceived betrayal by the third actor with three items each (e.g., “I feel betrayed by [name of the other user]”; α = .95) by using a 7-point Likert scale ranging from 1 (strongly disagree) to 7 (strongly agree). As control variables, we included perceived failure magnitude, attitude toward complaining, age, gender, prior experiences with Facebook, failure attributions, and the extent of customer contact in the participants’ daily work, all of which could affect customer reactions in failure situations (Hess, Ganesan, and Klein 2003; Pugh, Brady, and Hopkins 2018). We also measured self-efficacy and empathic concern as control variables to capture potentially relevant personal characteristics. Theme 3 in the Web Appendix provides all items, sources, and reliability estimates.

#### Analysis

To test mediated moderation, we ran three models (Baron and Kenny 1986; Muller, Judd, and Yzerbyt 2005). First, satisfaction was regressed on coalition (TA–SE, TA–CO), recovery (taking steps, apology), and their interaction. This established the unmediated coalition × recovery interaction (Muller, Judd, and Yzerbyt 2005). Second, perceived betrayal was regressed on coalition, recovery, and their interaction. We expected a positive effect of a TA–SE (vs. TA–CO) coalition on perceived betrayal. The other effects were included for comprehensiveness, since mediated moderation may also be based on the interaction during this step (Muller, Judd, and Yzerbyt 2005). Third, satisfaction was regressed on coalition, recovery, coalition × recovery, perceived betrayal, and perceived betrayal × recovery. We expected the perceived betrayal × recovery interaction to be significant, and the coalition × recovery interaction to be non-significant (or to reduce in effect; Baron and Kenny 1986; Muller, Judd, and Yzerbyt 2005). Comparing the third and the first model represents a hierarchical approach that reflects the proposed causal priority of the variables (Cohen et al. 2003).

Furthermore, we contrast-coded coalition (+.5 = TA–SE, −.5 = TA–CO) and recovery (+.5 = taking steps, −.5 = apology) and mean-centered perceived betrayal to meet the statistical requirements for the specified models (Muller, Judd, and Yzerbyt 2005). We included the control variables as independent variables in all regressions. Finally, we used percentile bootstrapping and a path model to estimate the combined effect of coalition→perceived betrayal and perceived betrayal × recovery→satisfaction (see Theme 4 in the Web Appendix).

### Results

#### Manipulation checks

To validate the coalition manipulation, the participants were asked to indicate who they thought the other user sided with by using a scale from 1 (the complainer) to 7 (Tesco), with 4 (nobody) as the neutral point. The participants correctly indicated that the other user took the complainer’s side (1.15) in the TA–CO coalition, Tesco’s side (6.56) in the TA–SE coalition, and nobody’s side (4.26) in the no-coalition condition (*F*[2, 523] = 1271.15, *p* < .001, η^2^ = .829). Post-hoc tests showed significant differences among the three groups (*p* < .001). The no-coalition group scored slightly over the scale midpoint, which was significant but minor in scope (*t*(174) = 3.97, *p* < .001, η^2^ = .022). To validate the recovery manipulation, the participants indicated how Tesco responded and correctly identified the recovery (i.e., no recovery, taking steps, or apology) in 96% of the cases; the recovery manipulation did not affect whom the participants thought the third actor sided with. The participants rated the scenarios as realistic (M = 5.76) on a scale from 1 (unrealistic) to 7 (realistic), which was higher than the scale midpoint in all conditions (*p*s < .001). The manipulations performed as intended.

#### Coalition, recovery, and satisfaction

We first examined the satisfaction ratings, as shown in Figure 3 (panel B). An analysis of covariance (ANCOVA) with the manipulations and control variables as independent variables revealed a main effect of recovery (*F*[2, 514] = 229.40, *p* < .001), such that no recovery yielded lower satisfaction ratings (1.81) compared to taking steps (4.41, *p* < .001) and apology (4.16, *p* < .001). It also indicated a main effect of coalition (*F*[2, 514] = 2.84, *p* = .060) and its interaction with recovery (*F*[4, 514] = 3.49, *p* = .008).

Simple analyses showed that taking steps yielded higher satisfaction ratings in the presence of the TA–SE coalition compared to the TA–CO coalition (4.82 vs 4.19, respectively; *F*[1, 112] = 6.05, *p* = .015, *d* = .46) and no coalition (4.82 vs 4.21, respectively; *F*[1, 112] = 4.67, *p* = .033, *d* = .41). An apology yielded higher satisfaction ratings in the presence of the TA–CO coalition compared to the TA–SE coalition (4.54 vs 4.06, respectively; *F*[1, 110] = 4.07, *p* = .046, *d* = .39) and no coalition (4.54 vs 3.88, respectively; *F*[1, 104] = 6.16, *p* = .015, *d* = .49). In addition, the TA–CO and TA–SE coalitions yielded similar satisfaction ratings when there was no recovery (*p* = .975), as did taking steps and apology when there was no coalition (*p* = .281), ruling out satisfaction differences due to the coalitions and recoveries only. We found no further effects.

#### Mediated moderation of perceived betrayal

Table 4, column Study 2, presents the results of the mediated moderation analysis. As expected, the results showed an interaction between coalition and recovery in Model 1 (β = .20, *p* = .002), and that the TA–SE compared to the TA–CO coalition increased perceived betrayal in Model 2 (β = .74, *p* < .001; H_4a_). Model 3 indicated a significant perceived betrayal × recovery interaction (β = .22, *p* = .024), while the coalition × recovery interaction was no longer significant (β = .05, *p* = .636). In addition, the joint effect of coalition→perceived betrayal and perceived betrayal × recovery→satisfaction was significant (β = .16, *p* = .024). The results supported perceived betrayal as a mediator of the moderation effect of coalition. Figure 3, panel C, shows the perceived betrayal × recovery interaction. As perceived betrayal increased, taking steps yielded larger and apology lower satisfaction values. For values ≤1.49, the effect of apology was larger than that of taking steps, and for values ≥3.76, the effect was the opposite, supporting H_4b_ and H_4c_.

**Table 4.** Mediated Moderation Results of Study 2 and Study 3.

	Expected Direction	Study 2: Tesco						Study 3: Zara					
		(1) Satis-Faction		(2) Perceived Betrayal		(3) Satis-Faction		(1) Satis-Faction		(2) Perceived Betrayal		(3) Satis-Faction	
		Beta	*p*	Beta	*p*	Beta	*p*	Beta	*p*	Beta	*p*	Beta	*p*
Unmediated moderation effects													
Coalition (TA–SE)^a^	Model 2, H_4a_: +	.02	.791	.74	<.001	−.01	.939	.09	.070	.84	<.001	−.01	.900
Recovery (taking steps)^a^		.06	.399	.02	.614	.06	.387	−.36	<.001	−.03	.329	−.36	<.001
Coalition × recovery	Model 1^b^: +	.20	.002	.07	.134	.05	.636	.13	.012	−.01	.667	−.04	.663
Mediated moderation effects													
Perceived betrayal		—	—	—	—	.01	.959	—	—	—	—	.13	.198
Perceived betrayal × recovery	Model 3, H_4b,c_: +	—	—	—	—	.22	.024	—	—	—	—	.20	.034
Control variables													
Age		−.07	.344	.02	.726	−.07	.296	.02	.704	.05	.102	.01	.825
Gender (male)^c^		.02	.778	.02	.630	.04	.525	−.10	.076	.00	.925	−.08	.130
Gender (no disclosure)^c^		−.03	.687	.06	.166	−.04	.554	−.03	.504	.01	.682	−.04	.448
Failure magnitude		−.11	.124	−.12	.014	−.11	.131	−.07	.199	.12	<.001	−.09	.092
Failure attributions		−.40	<.001	.12	.012	−.38	<.001	−.23	<.001	.09	.004	−.23	<.001
Attitude toward complaining		.02	.745	−.01	.892	.05	.499	.11	.037	.01	.759	.12	.032
Prior experiences^d^		.07	.310	−.07	.115	.06	.415	−.07	.224	.01	.824	−.07	.201
Customer contact in daily work		−.09	.174	.01	.869	−.10	.124	−.07	.186	.02	.521	−.08	.142
Self-efficacy		.02	.755	.00	.987	.03	.659	.01	.891	−.01	.814	.00	.955
Empathic concern		−.03	.695	.04	.415	−.02	.801	.12	.041	−.07	.031	.15	.012

^a^Coalition: +.5 = TA–SE, −.5 = TA–CO; recovery: +.5 = taking steps, −.5 = apology.

^b^No hypothesis formulated.

^c^Comparing male and “do not wish to disclose” answer options with female as reference category.

^d^Prior experiences with Facebook and fashion stores for Study 2 and Study 3, respectively.

*Notes*: Recovery, firm recovery; perceived betrayal, complainers’ perceived betrayal by the third actor; satisfaction, complainers’ satisfaction with the recovery by the firm.

### Discussion of Study 2

In Study 2, we examined how a TA–SE (vs. TA–CO) coalition shifts the recovery preferences of the complainer. As expected, the complainers felt betrayed when the third actor sided with the service employee (compared to the complainer), consistent with H_4a_. The complainers’ feelings of betrayal by the third actor, in turn, impacted their satisfaction with the firm’s recovery, strengthening the recovery effect of taking steps and weakening that of apology, consistent with H_4b_ and H_4c_. Complementing Study 1, the results revealed coalition effects on the relation level, showing an effect cascade that connected the service employee–third actor relation (coalition) to the complainer–service employee relation (shift in recovery preferences) through the third actor–complainer relation (perceived betrayal). The results also supported the notion of broadening the scope of perceived betrayal, which so far has largely focused on complainers’ perceived betrayal by the service firm. We conceptualized perceived betrayal by focusing on the third actor in the multi-actor structure and found that it explains the moderation effect of a coalition. For managers, the satisfaction ratings for the hypothesized conditions indicated upward deviations from the otherwise average ratings, highlighting that adapting to the coalitions can improve recovery effectiveness. We designed Study 3 on these findings by offering a replication with different adjustments, as outlined next.

## Study 3

### Method

#### Experimental design

To broaden the scope of Study 2 findings and assess their robustness, we added three variations in Study 3. It comprised an offline setting to expand the findings beyond the previous online context. The third actor represented a friend, rendering a stronger tie between the complainer and the third actor than before. We assessed whether the proposed shift in recovery preferences would occur when taking steps and apology are components within a multi-component recovery approach. This study used a 2 (coalition: TA–CO coalition, TA–SE coalition) × 3 (recovery: compensation only, compensation plus taking steps [i.e., taking steps], compensation plus apology [i.e., apology]) between-subjects design. We did not include a no-coalition condition, since our aim was to replicate Study 2 results. We used an apparel shopping episode because it represents an under-researched setting (Khamitov, Grégoire, and Suri 2020) and chose Zara as a well-recognized firm in the United Kingdom.

The participants read a scenario of a customer who went to Zara to shop for summer apparel together with a friend. The customer was charged the full price for a jacket, although it had been promoted with a 20% discount. Upon realizing the mistake, the customer told the friend of their intention to complain to the store, and we manipulated the friend’s reply as “I feel for you; Zara needs to pay more attention. Such failures are really annoying not only from a financial point of view but as a matter of principle too” (TA–CO coalition) or “Move on; this is not the end of the world. The price is low; just be grateful for the good deal that you got from Zara, which also cannot offer discounts for every item in store” (TA–SE coalition). After that, the participants read the store manager’s response, explaining that the jacket was wrongly promoted and offering a 20% discount on the original price (compensation only), a 20% discount plus taking steps (i.e., ensuring that there are no other wrongly promoted items and discussing this issue with the quality department), or a 20% discount plus an apology (apology). Theme 2 in the Web Appendix provides the scenarios.^5^

#### Data collection, measures, and analysis

Data collection, measures, and analysis were the same as those in Study 2, with some adaptations. Data were gathered in spring 2022 from the crowdsource panel Prolific, offering each subject 80 pence sterling for participation. Upon completion, we excluded 11 of the 517 respondents because they had provided suspicious answers. The final sample included 506 United Kingdom residents (mean age 38.6 years, 49.2% female), with cell sizes ranging from 81 to 87. The items for measuring satisfaction were adapted to the new context of measuring satisfaction with complaint handling.

### Results

#### Manipulation checks

Manipulation checks similar to those used in Study 2 indicated that all of our manipulations performed as intended.^6^ The participants rated the scenarios as realistic (M = 6.03) and above the scale midpoint in all conditions (*p*s < .001).

#### Coalition, recovery, and satisfaction

Figure 3, panel B, depicts the satisfaction cell means. An ANCOVA with the manipulations and the control variables as the independent variables showed a main effect of recovery (*F*[2, 491] = 32.88, *p* < .001), and simple analyses indicated higher satisfaction ratings in the apology condition (6.13) compared to all other conditions (*p*s < .001).^7^ The ANCOVA also revealed a main effect of coalition (*F*[1, 491] = 4.62, *p* = .032) and its interaction with recovery (*F*[2, 491] = 2.70, *p* = .068).

Simple analyses for the taking steps and apology conditions illustrated the coalition × recovery interaction more clearly (*F*[1, 320] = 6.41, *p* = .012). The taking steps condition yielded higher satisfaction ratings in the presence of the TA–SE compared to the TA–CO coalition (5.48 vs 4.95, respectively; *F*[1, 156] = 6.86, *p* = .010, *d* = .42), and the apology condition yielded similar satisfaction ratings in the presence of both TA–CO and TA–SE coalitions (6.18 vs 6.08, respectively; *F*[1, 156] = 0.40, *p* = .528, *d* = −.11). In addition, the satisfaction ratings in the compensation-only condition did not differ from each other (*p* = .308).

#### Mediated moderation of perceived betrayal

The results, shown in Table 4 (column Study 3), indicated an interaction between coalition and recovery in Model 1 (β = .13, *p* = .012), and that the TA–SE (vs. TA–CO) coalition increased perceived betrayal in Model 2 (β = .84, *p* < .001; H_4a_). In Model 3, we found a significant perceived betrayal × recovery interaction (β = .20, *p* = .034), while the coalition × recovery interaction was no longer significant (β = −.04, *p* = .663). In addition, the joint effect of coalition→perceived betrayal and perceived betrayal × recovery→satisfaction was significant (β = .17, *p* = .027). The results supported the proposed mediated moderation. Figure 3, panel C, plots the perceived betrayal × recovery interaction. As perceived betrayal increased, taking steps yielded larger satisfaction values, while the effect of apology decreased only slightly, the difference being significant for values ≤5.43 in perceived betrayal. These results supported H_4b_ but not H_4c_.

### Discussion of Study 3

The objective of Study 3 was to replicate Study 2 results. For this purpose, we used an offline setting in Study 3, a strong tie between the complainer and third actor, and a multi-component recovery approach in which taking steps and an apology were offered alongside a compensation. The TA–SE (vs. TA–CO) coalition increased the complainer’s perceived betrayal by the third actor, as we expected in H_4a_, which strengthened the recovery effect of taking steps, consistent with H_4b_. Contrary to H_4c_, increased levels of perceived betrayal did not weaken the recovery effect of an apology. This result remains inconclusive and might have stemmed from individuals generally expecting an apology in such a shopping context.

## General Discussion

Recently, a small body of research on triads in the service failure and recovery domain has started to investigate social dynamics in service failure situations (Table 1). We expand this body of work and introduce coalitions as an impactful phenomenon, contributing theoretically and managerially to the literature in three ways.

### Theoretical Implications

As a first contribution, we demonstrate that coalitions are a pervasive phenomenon and provide insights into the effect pattern in the triad (Study 1). We found that coalitions occurred in 32.0% (±3.5%) of the complaint episodes on retailers’ official Facebook pages. The complaint episodes expressed a slightly negative affective tone (45.0; neutral = 50), which deteriorated to a more negative tone in the range of 30.44–37.80 in the presence of a coalition. The effect sizes were absolute in a .20 to .41 range. Smaller effect sizes were expected, as they were based on actual behaviors. The coalition-induced negative shift of the affective tone of the triad supports the idea that coalitions create an opinion imbalance, yielding tensions that are reflected in the greater negative attitudes of the triad members (Neubaum and Krämer 2017; Sherif et al. 1951). Our study of coalitions expands the scope of the triadic structures examined to date in that the analyzed triad was closed and considered the third actor as multiple individuals (Table 1).

In terms of the effect pattern, both the TA–CO and TA–SE coalitions led to a more negative affective tone of the triad. The effects of both coalitions were not additive in that their joint effect did not exceed their individual effects. This suggests a potential lower limit to the downward trajectory of coalitions. Possibly, the triad members engaged in selective information processing, disregarding an opposing standpoint (Jeong et al. 2019). We obtained two unexpected findings. The TA–CO coalition yielded a larger negative shift, which indicates a collective rumination about the failure. The TA–SE coalition acted as a partial buffer, reducing the negative impact of the TA–CO coalition. While this buffering effect requires some caution (*p* = .071), it indicates that other customers defending the retailer are somewhat successful. Given that our findings focus on the triad as a unit of analysis and use field data, they may inform other failure situations that can be represented by the examined triad (i.e., an individual complainer and service employee plus one or multiple others), such as a group of friends visiting a restaurant, a gym class, or corresponding experience-sharing on social media channels.

As a second contribution, we show that the TA–SE (vs. TA–CO) coalition increased complainers’ feelings of betrayal by the third actor, which impacted complainers’ satisfaction with the firm’s recovery in that it strengthened the recovery effect of taking steps and weakened that of an apology (Studies 2 and 3). This finding complements our results obtained for the triad by revealing coalition effects for the triad relations. Having conceptualized the triad as a structure of connected relations, the coalition-induced change in recovery preferences shows one way in which the relations in the triad are connected, reflecting an effect cascade from the service employee–third actor (coalition) to the complainer–service employee (shift in recovery preferences) through the third actor–complainer (perceived betrayal) relation.

Our results also expand prior research on triads (Table 1). First, we uncover the explanatory value of perceived betrayal and offer a novel conceptualization that focuses on betrayal by the third actor. This suggests broadening its applicability to multi-actor structures, speaking to efforts for an integrated framework of process variables (e.g., Khamitov, Grégoire, and Suri 2020). Second, this study enhances the small body of research on triads. By exploring how two or more relations are connected, this stream of research offers novel insights into social dynamics (i.e., conduit, initiation, and coalition effects; Figure 1) and, as such, provides a promising angle for understanding multiple co-existing relations (Khamitov, Grégoire, and Suri 2020). Finally, while mediated moderations seem underutilized in the research stream, our findings illustrate their value for a better understanding of moderation effects.

### Managerial Implications

Firms are likely to be confronted with failure situations in which others take sides with the complainer or the service employee. Such coalitions present a challenge as they occur outside of a firm’s sphere of influence. However, research-based insights to guide managers about the prevalence of coalitions, the nature of their effects, and potential remedial strategies are lacking. As a third contribution, this article addresses two key managerial questions.

How pervasive are coalitions and are their effects always harmful? We find that coalitions occur in approximately one-third of the complaints made on retailers’ official Facebook pages and that the presence of a coalition yields a substantial downward trajectory of 16%–32%, shifting the affective tone of the online conversations from neutral to negative. Both types of coalitions—others siding with the complainer (TA–CO) or the service employee (TA–SE)—are harmful. Yet, there is also room for encouragement. The joint impact of the two coalitions is not more harmful than that of each coalition individually. Moreover, the TA–SE coalition tends to partially mitigate the comparably larger impact of the TA–CO coalition.

How do coalitions interfere firm responses and when are they effective? In our sample, the retailers responded between 0% (e.g., Asda) and over 67% (e.g., Tesco) of times when a complaint was made on Facebook. Coalitions render firm responses that aim to create a more positive affective tone in online conversations ineffective and shift complainers’ recovery preferences. Given that triads remain under-researched, our results corroborate that, in the absence of a coalition, personal and positive response styles, as well as accommodative response content (taking steps, apology, and explanation), generate a more positive affective tone. Moreover, firm responses may mitigate some of the detrimental impact of subsequent coalitions. An adaptive recovery approach may partially mitigate the coalitions’ impact on online conversations; we suggest firms to emphasize a speedy response that conveys accommodative content in a personal and positive manner. To improve the recovery effectiveness for complainers, firms should use taking steps when others side with the service employee and an apology when others side with the complainer, either employed as stand-alone recovery or as part of a multi-component approach. When complainers feel betrayed by others who side with the firm, a factual recovery allows complainers to shift their focus away from the emotional processing of the betrayal, while a socio-emotional recovery may rather prolong it.

### Limitations and Future Research

Based on the limitations of our work, Table 5 provides six areas for future research. We analyzed a typical triad, yet various other compositions are conceivable and should be explored, such as when a robot is the service agent and another service employee is a third actor (area 1). While the present research provides a first analysis of coalitions, they are a rich phenomenon with various unexplored facets (area 2). For example, future research may analyze how one coalition leads to subsequent coalitions, and whether the impact differs in relation to the type of coalitions (e.g., protective vs. predatory; Vedel, Holma, and Havila 2016). We found that coalitions impair the positive effect of firm responses on the affective tone of the triad, questioning whether alternative response strategies, such as humor (Béal and Grégoire 2022), can disrupt harmful coalition dynamics (area 3). In addition, an expansion of consumption settings is desirable (e.g., virtual realities; area 4). While our results portrayed largely negative consequences, positive effects are also conceivable and should be researched (area 5). Finally, the findings may inspire research in other fields, for instance, to explore the effects of multiple touchpoints on customer experiences, an aspect inherent to coalitions in triads (area 6).

**Table 5.** A Research Agenda.

Areas	Research Gap and Research Questions
1. Triad members and consequences	*Research gap:* The analyzed composition of the triad (an individual complainer, a service employee, and one or multiple others) and the focus on the consequences for the complainer formed a starting point for the analysis of coalitions. *Research questions:* How do coalition dynamics unfold when there are deliberate power differences among the triad members (e.g., the third actor is another service employee, the complainer is represented by a group of customers experiencing the same failure)? How do coalition dynamics change when actors take non-human forms, such as service robots, brands, and even nature as an entity? Finally, what consequences do coalitions have for the other actors involved, besides the complainer; the service employee being of particular relevance, for instance?
2. Nature of coalitions	*Research gap:* Coalitions are a rich phenomenon. Thus, questions remain in the context of service failures. *Research questions:* How do more complex and multiple coalitions occur? Could there be a ripple effect with one coalition triggering another? Are there further ways in which the relations are connected and coalition effects impact the triad members? Given that coalitions differ in type (e.g., being protective or predatory in nature), is it possible to identify different coalition types with idiosyncratic effects? While having found effects on an affective level, do coalitions also operate on a cognitive level? Finally, which other mechanisms besides complainers’ perceived betrayal by the third actor can explain coalition effects (e.g., the emotional support, rather than betrayal, by the third actor)?
3. Coalitions and firm responses	*Research gap:* Once a coalition is formed, firm responses became ineffective in restoring the affective tone of the triad. Thus, different questions remain on how to tackle coalition dynamics. *Research questions:* Which recovery strategies and response styles are effective in making a positive change in the triad after a coalition? When considering coalitions as a detrimental dynamic, how can firms prevent coalitions in the first place and, once formed, curtail the formation of further coalitions? Could alternative response strategies, such as humor (Béal and Grégoire 2022), disrupt coalition dynamics?
4. Coalitions across consumption settings	*Research gap:* The present research covered field data across different failure instances and retailers. Yet, consumption settings vary beyond what we analyzed, offering room for future research. *Research questions:* Are effects similar for offline contexts, for example, where consumption is marked by multiple actors (e.g., hospitality, travel, and medical consultations), hybrid and other virtual settings (e.g., chatting with friends while shopping, virtual reality), and contexts that contain hedonic versus utilitarian elements (e.g., Johnen and Schnittka 2019)? Given that culture shapes social interactions, how does culture interfere with coalitions?
5. Positive sides of coalitions	*Research gap:* While coalitions are discussed as a pervasive phenomenon (Komorita and Chertkoff 1973), there may also be positive sides to them. Thus, questions related to coalition consequences and motives are worth exploring. *Research questions:* Could coalitions exert positive effects, similar to the less intuitive, buffering effect seen in the TA–SE coalition? Furthermore, is the formation of coalitions possibly driven by motives that are not negative, such as when complainers fear being unsuccessful with their complaint?
6. Broader considerations	*Research gap:* Examining coalitions in triadic structures yielded novel and rich insights for the service failure and recovery literature. It appears fruitful to expand the concepts of coalitions and triads to other fields of inquiry. *Research questions:* Do coalitions cause similar effects for other transgression types (e.g., customer incivility) and negative events (e.g., brand transgressions and product-harm crises)? Given that triads and coalitions focus on multiple touchpoints, how can we advance our understanding of the effects of multiple touchpoints from a customer experience perspective (Lemon and Verhoef 2016)? Finally, which insights can we gain from an expansion to four or more actors?

## Supplemental Material


Supplemental Material - Coalitions and Their Negative Consequences: An Examination in Service Failure-Recovery Situations
Supplemental Material for Coalitions and Their Negative Consequences: An Examination in Service Failure-Recovery Situations by Holger Roschk, Masoumeh Hosseinpour, and Jan Breitsohl in Journal of Service Research

Supplemental Material - Coalitions and Their Negative Consequences: An Examination in Service Failure-Recovery Situations
Supplemental Material for Coalitions and Their Negative Consequences: An Examination in Service Failure-Recovery Situations by Holger Roschk, Masoumeh Hosseinpour, and Jan Breitsohl in Journal of Service Research

## Notes

1. Another possible distinction between association and connectedness may be whether the third actor plays a passive or active role in the exchange. Association is about the existence of relations, and the third actor can remain passive (e.g., an observer). For connectedness, the third actor likely needs to be more active, given that one relation is required to influence another. However, caution is needed since this distinction may not hold true for every conceivable situation. Therefore, the key distinction between association and connectedness should be seen in whether one relation influences another, an aspect that is not part of association but is the defining criterion for connectedness.

2. Realistic group conflict theory in this context would assume that the service employee, much like the other triad members, has an underlying fear of creating minority group situations. While service employees may not always be considered in-group members, reaching an opinion congruence in a service failure setting will likely still be desirable by all triad members.

3. Taking steps is a broader version of credibility feedback in that it does not focus on rectifying the cause of the failure but more broadly concentrates on solving the problem (Van Vaerenbergh et al. 2019). We excluded compensation from our considerations because it can backfire in online settings (Herhausen et al. 2019).

4. We used a one-tailed test when comparing two HLM coefficients since we tested directional expectations.

5. We pre-tested the failure magnitude (*N* = 60; same items as in Study 2), varying the promoted discount rate across 10%, 20%, and 30%. The failure magnitudes were 4.59, 5.26, and 5.40, respectively. Since the 20% and 30% conditions differed from the 10% condition (*p* = .032) but not from each other (*p* = .711), we chose a rate of 20%. We also pre-tested the compensation amount (*N* = 20). An apology and partial compensation of 10% yielded an average satisfaction of 2.98, which was below the scale midpoint (*p* = .009, *d* = 0.73). This indicated that the participants expected the originally (wrongly) promoted 20% discount.

6. The participants indicated that the friend took the complainer’s side (1.18) in the TA–CO coalition and Zara’s side (6.31) in the TA–SE coalition (*F*[1, 500] = 3929.65, *p* < .001, η^2^ = .887). To validate the recovery manipulation, we asked the respondents if the store manager promised to verify the sales prices and apologized (one item each: 1 = strongly disagree, 7 = strongly agree). The group means differed, as intended, across the compensation-only, taking steps, and apology conditions for the taking steps item (1.47, 6.49, and 1.47, respectively; F(2, 500) = 1843.71, *p* < .001, η^2^ = .881) and the apology item (2.98, 3.35, and 6.77, respectively; F(2, 500) = 269.86, *p* < .001, η^2^ = .519). The post-hoc tests showed no differences apart from the intended recovery. The fact that the apology ratings do not approach the lower end of the scale can be attributed to compensation as a baseline recovery. The coalition manipulation did not influence the manipulation checks for recovery, and vice versa.

7. Given that the satisfaction ratings did not differ in the compensation only condition, the taking steps condition when there was a TA–SE coalition (5.48) yielded higher satisfaction ratings than the compensation only condition (5.12, *p* = .049).
