# Supplementary material for: Coalitions and Their Negative Consequences: An Examination in Service Failure-Recovery Situations
Source: J Serv Res. 2023 Mar 30;26(4):614–35. doi: 10.1177/10946705231163884 (PMC10522451; doi:10.1177/10946705231163884)
Supplement: Supplemental Material - Coalitions and Their Negative Consequences: An Examination in Service Failure-Recovery Situations [file sj-pdf-1-jsr-10.1177_10946705231163884.pdf]

Coalitions and Their Negative Consequences:

An Examination in Service Failure-Recovery Situations

WEB APPENDIX

Contents

Theme 1: Statistical Details on the Hierarchical Linear Models for Study 1

Theme 2: Experimental Materials for Studies 2 and 3

Theme 3: Constructs, Items, and Sources for Studies 2 and 3

Theme 4: Alternative Test of Mediated Moderation Based on Path Analysis

Theme 1: Statistical Details on the Hierarchical Linear Models for Study 1

This theme provides the formulaic representation of the hierarchical linear models (HLMs) used in Study 1. The data has a nested structure, where individual data points (user comments) belong to the same higher order structure (complaint episode). HLMs are specifically designed for such data (Field 2018). For this purpose, they comprise multiple models for the different levels on which the data is structured.

We used two-level HLMs, where the Level 1 model specifies the relationships at the individual level (user comments), and the Level 2 model at the higher order level (complaint episode). The HLM model for the impact of both coalitions on affective tone (Model 1 from Table 3 in the main text) has the following Level 1 equation:

$$AT_{ij} = \beta_{0j} + \beta_{1j}TACO_{ij} + \beta_{2j}TASE_{ij} + \beta_{3j}TACOxTASE_{j} + \beta_{4j}WOC_{ij} + \beta_{5j}SLW_{ij} + \beta_{6j}CPL_{ij} + \beta_{7j}FRM_{ij}$$

AT denotes the affective tone of comment i in the complaint episode j, β_{0j} represents the intercept, β_{1j} … β_{7j} are the slopes for the Level 1 variables presence of a TA–CO coalition (TACO), presence of a TA–SE coalition (TASE), their interaction as product term (TACOxTASE), word count (WOC), percentage of words with more than six letters (SLW), complaint comment (CPL), and firm comment (FRM).

In the Level 2 model, the intercept β_{0j} from the Level 1 becomes the dependent variable to incorporate the hierarchical data structure and the Level 2 variables. The equations are:

$$\beta_{0j} = \gamma_{00} + \gamma_{01}FCO_{j} + \gamma_{02}FST_{j} + \gamma_{03}OFA_{j} + \gamma_{04}FRE_{j} + \gamma_{05}FSE_{j} + \gamma_{06}MED_{j} + \gamma_{07}BCO_{j} + \gamma_{08}BFF_{j} + \gamma_{09}BGR_{j} + \gamma_{010}BBU_{j} + \gamma_{011}DAY_{j} + u_{0j},$$

$\beta_{1j} = \quad \gamma_{10}$,

...

$\beta_{7j} = \quad \gamma_{70}$.

The parameter u_{0j} denotes the complaint episode membership, modelling that the comments originated from different complaint episodes (i.e., random intercept, fixed slope model). The parameters γ_{01} … γ_{011} are the slopes of the Level 2 variables failure controllability (FCO), failure stability (FST), outcome failure (OFA), failure reversibility (FRE), mediators (MED), branch: coffee chains (BCO), branch: fast-food (BFF), branch: general retailers (BGR), brand buzz (BBU), and daytime (DAY). For calculation, we used HLM software 8.02 with full maximum likelihood estimation, as often used in consumer research (e.g., Martin and Hill 2012).

The HLM for Model 2 from Table 3 in the main text follows the same specification, and additionally comprises presence of a firm response as Level 1 variable. The HLM for Model 3 from Table 3 in the main text also follows the same specification as Model 1, and additionally includes two variables for the presence of a firm response depending on whether a coalition was absent or present.

Additional References for Theme 1 of the Web Appendix

Field, Andy (2018), *Discovering Statistics Using IBM SPSS Statistics,* 5th ed. Thousand Oaks, CA: Sage.

Martin, Kelly D., and Ronald P. Hill (2012), "Life Satisfaction, Self-Determination, and Consumption Adequacy at the Bottom of the Pyramid," *Journal of Consumer Research*, 38 (6), 1155–1168.

Theme 2: Experimental Materials for Studies 2 and 3

Experimental Materials Study 2

FIGURE W1
STUDY 2 SCENARIOS AND CONDITIONS

1 Failure and Complaint

Please imagine you are Peter Moore. Peter regularly buys Butternut Squash baby food from Tesco supermarket, and follows Tesco on Facebook. However, recently, his young daughter got sick after consuming a babyfood product from Tesco, and he decides to voice his dissatisfaction to Tesco on Facebook, as depicted below:

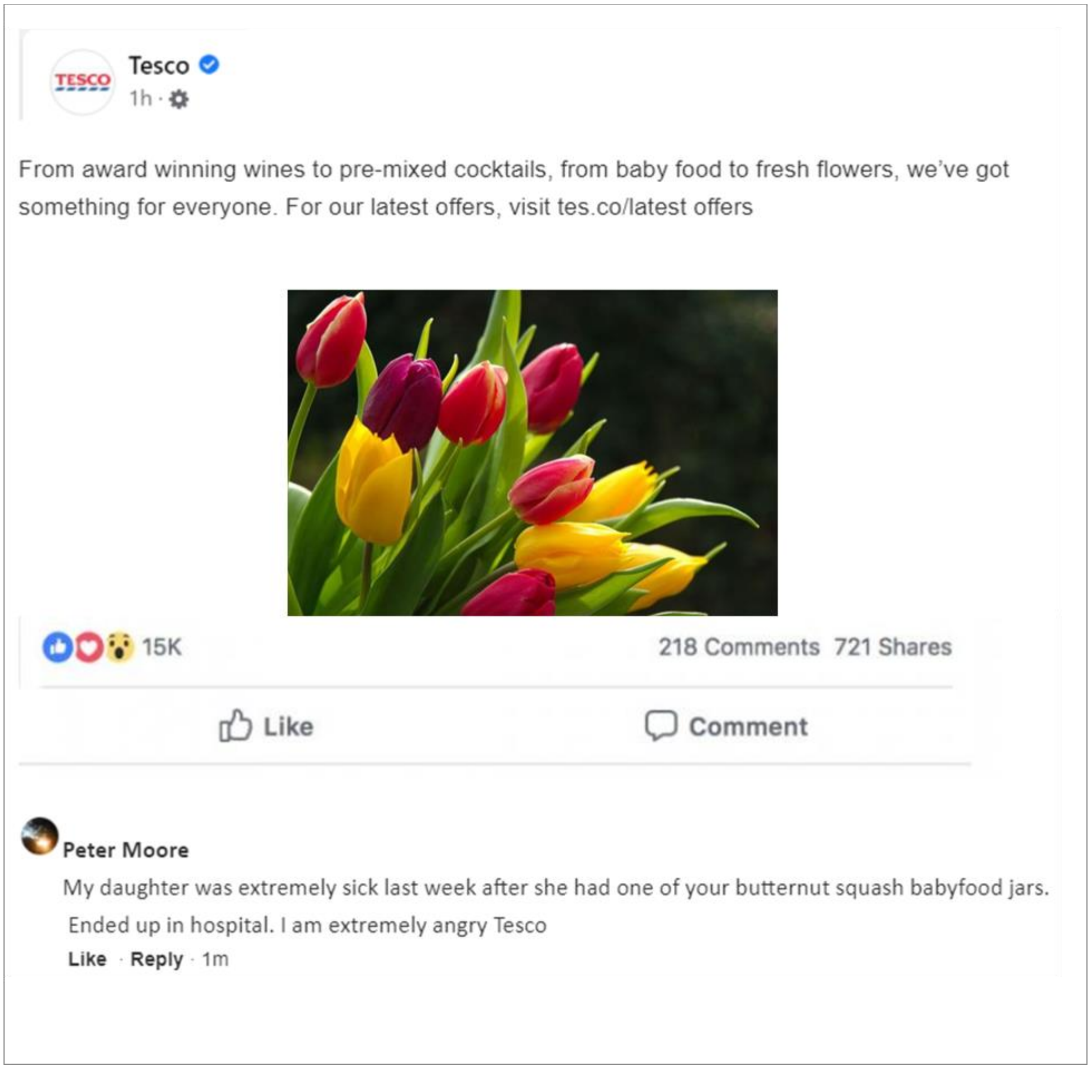

2 Coalition Conditions

Again, please imagine you are Peter Moore, and that you read the following comment from another consumer below your own:

TA-CO coalition:

Peter Moore
My daughter was extremely sick last week after she had one of your butternut squash babyfood jars. Ended up in hospital. I am extremely angry Tesco
Like · **Reply** · 3m

Babs Dowell
@Peter Moore – I would feel the same mate. Tesco needs to be more transparent, this sounds really serious.
Like · **Reply** · 1m

TA-SE coalition:

Peter Moore
My daughter was extremely sick last week after she had one of your butternut squash babyfood jars. Ended up in hospital. I am extremely angry Tesco
Like · **Reply** · 3m

Babs Dowell
@Peter Moore – Move on, your child might be sick from something else. I buy Tesco's baby food all the time, never had a bad experience.
Like · **Reply** · 1m

No coalition:

Peter Moore
My daughter was extremely sick last week after she had one of your butternut squash babyfood jars. Ended up in hospital. I am extremely angry Tesco
Like · **Reply** · 3m

Babs Dowell
@Tesco, do you have more information on the Wine offers?
Like · **Reply** · 1m

3 Recovery Conditions

When you check the Facebook page again a little later, you find the following response from Tesco:

Apology:

Author
Tesco

Dear **Peter Moore**. I'm very sorry that your son has been sick. I hope he will be better soon and I can understand why this was upsetting. If you are happy to bring the product into one of our stores, we would be happy to examine it for you. We are very sorry that you have had such a negative experience.

Kindest regards,

Kayley - Customer Care

Like · **Reply** · 1m

Taking steps:

Author
Tesco

Dear **Peter Moore**. As a precautionary measure, we would advise not to use these baby food jars. If you send us the jars, or bring it to the store, we will carefully examine them with our quality control team, test the product in our lab and based on the results ensure that all our products are of the high quality that our customers are used to and expect from us. We will also get back in touch with you to discuss with you directly how we can make it up to you.

Kindest regards,

Kayley - Customer Care

Like · **Reply** · 1m

When you check the Facebook page again a little later, you notice some more comments but you do not find a response from Tesco to the baby food complaint:

No recovery:

Morven Faulkner

Can anyone tell me where to find your offers for pre-made cocktails – have they removed them?

Like · **Reply** · 1m

Eleni Baklava

@Jan Tomasik – have you seen this offer pal?

Like · **Reply** · 3m

Ulrich Rice

Ulrich Reich: When will you open a store in the Greenwood region?

Like · **Reply** · 9m

Experimental Materials Study 3

Table W1. Study 3 Scenarios and Conditions.

In the following we describe a shopping episode. Please imagine you are Anne, who is shopping for some summer clothes together with her close friend Sebastian.[a]

1 Failure and Complaint

Anne went to Zara to shop for some summer clothes. It was a special occasion for her because the last weeks were quite busy and the shops had various sales running.

To make the shopping trip more enjoyable, her close friend Sebastian accompanied Anne. After browsing the store, Anne found a jacket, a shirt, and a pair of trousers which were on sale with a 20% discount. Anne was fond of the jacket. She paid, and upon leaving the store, Anne checked the receipt and realized that she was charged the full price for the jacket.

2 Coalition Conditions

When she realized that an error had been made, Anne told Sebastian that she would go to complain to the store manager.

TA–CO coalition:	*TA–SE coalition:*
Sebastian was supportive, saying to Anne: “I feel for you; Zara needs to pay more attention. Such failures are really annoying not only from a financial point of view but as a matter of principle too.”	Sebastian was not supportive, saying to Anne: “Move on; this is not the end of the world. The price is low; just be grateful for the good deal that you got from Zara, which also cannot offer discounts for every item in store.”

3 Recovery Conditions

After Anne complained, the store manager said to Anne:

Compensation plus apology [i.e., apology]:	*Compensation plus taking steps [i.e., taking steps]:*	*Compensation only:*
“I am truly sorry for what has happened. We experienced problems with the system the whole day, which was probably the reason why the jacket was wrongly promoted with the discount. I can fully understand why this would be upsetting to you and can offer you a 20% discount on the jacket’s regular price. Once again, I am really sorry that you had this negative experience, please accept my sincere apologies.”	We experienced problems with the system the whole day, which was probably the reason why the jacket was wrongly promoted with the discount. I will have a sales assistant thoroughly double check that there are no other cases like this in the store. Moreover, I will discuss this with our quality control team to assess how we can prevent such problems in the future. I can offer you a 20% discount on the jacket’s regular price.”	“We experienced problems with the system the whole day, which was probably the reason why the jacket was wrongly promoted with the discount. I can offer you a 20% discount on the jacket’s regular price.”

[a]We switched the gender of the complainer and third actor from Study 2 and left that of the store manager unspecified, given the objective to examine robustness of Study 2 results.

Theme 3: Constructs, Items, and Sources for Studies 2 and 3

Table W2. Constructs, Items, and Sources for Studies 2 and 3.

Transaction-specific satisfaction[a] (α = .97/.98, *M* = 3.50/5.49, SD = 1.95/1.39[b]; Pugh, Brady, and Hopkins 2018)
- Judging this particular service encounter, I am satisfied.
- Judging this particular service encounter, I am pleased.
- This specific service encounter was favorable.

Perceived betrayal by the third actor (α = .95/.95, *M* = 3.37/2.91, SD = 1.74/1.97; Grégoire, Tripp, and Legoux 2009)
- How do you feel about the reaction of [name of the third actor] to you? …
- … I feel betrayed by her/him.
- … She/He let me down.
- … I feel cheated by her/him.

Failure magnitude (α = .89/.86, *M* = 6.26/5.28, SD = 0.99/1.19; Hess, Ganesan, and Klein 2003)
- For me as a customer, the failure …
- … is significant.
- … is major.
- … is severe.
- … causes a lot of inconvenience.

Failure attributions (*M* = 4.78/5.09, SD = 1.65/1.37; Nazifi et al. 2021)
- There are actions that Tesco/Zara could have taken, but did not take to prevent the failure.

Attitude toward complaining (*M* = 4.32/3.64, SD = 1.78/1.76; Roschk and Gelbrich 2014)
- I am usually reluctant to complain in a service encounter. (R)

Prior experiences with Facebook/fashion stores (*M* = 5.04/4.24, SD = 1.97/1.75; Hess, Ganesan, and Klein 2003)
- I regularly visit Facebook/fashion stores.

Customer contact in daily work (*M* = 4.67/4.25, SD = 2.00/2.21)
- In my own job, I regularly interact with customers.

Self-efficacy (*M* = 5.14/5.02, SD = 1.27/1.30; Chen, Gully, and Eden 2001)
- In life, I will be able to achieve most of the goals that I have set for myself.

Empathic concern (*M* = 6.03/6.01, SD = 0.92/0.89; Davis 1980)
- I would consider myself a good-hearted person.

Age (*M* = 37.92/38.59, SD = 11.81/13.77)
- How old are you? (in years)

Gender[c] (*M* = 0.60/0.50, SD = 0.49/0.50)
- You are: male (0), female (1).

[a]Transaction-specific satisfaction in Study 3 used the following items (Hess, Ganesan, and Klein 2003; Roschk and Kaiser 2013): "I am happy with how my complaint is being handled," "In my opinion, the store manager provided a satisfactory response to my problem," "I am satisfied with the handling of my complaint," "I am satisfied with how the store manager handled the problem on this particular occasion."
[b]Cronbach's α, mean, and standard deviation for Study 2/Study 3.
[c]Numbers without the additional response option "do not wish to disclose," which was used 0.9% and 0.8% in Study 2 and 3, respectively.
Notes: All items were measured along a seven-point Likert-type scale from 1 (strongly disagree) to 7 (strongly agree), unless for age and gender. (R), reverse-coded item.

Additional References for Theme 3 of the Web Appendix

Chen, Gilad, Stanley M. Gully, and Dov Eden (2001), "Validation of a New General Self-Efficacy Scale," *Organizational Research Methods*, 4 (1), 62–83.

Davis, Mark H. (1980), “A Multidimensional Approach to Individual Differences in Empathy,” *Journal Supplement Abstract Service: Catalog of Selected Documents in Psychology*, 10 (4), 85–103.

Grégoire, Yany, Thomas M. Tripp, and Renaud Legoux (2009), “When Customer Love Turns into Lasting Hate: The Effects of Relationship Strength and Time on Customer Revenge and Avoidance,” *Journal of Marketing*, 73 (6), 18–32.

Roschk, Holger and Susanne Kaiser (2013), “The Nature of an Apology: An Experimental Study on how to Apologize After a Service Failure,” *Marketing Letters*, 24 (3), 293–309.

Theme 4: Alternative Test of Mediated Moderation Based on Path Analysis

The mediated moderation in the main text refers to the main effect of coalition on perceived betrayal which moderates the effect of recovery on satisfaction (Muller et al. 2005). Alternatively, such a constellation could also be tested by using a path model approach (van Kollenburg and Croon 2022). The path model depicted in Figure W2 combines Models 2 and Models 3 from Table 4 in the main text, thus directly testing our conceptual framework. Moreover, it allows us to test the combined effect of coalition→perceived betrayal and perceived betrayal × recovery→satisfaction. The statistical model shown in Figure 2 thus represents the moderated mediation shown in Figure 2, panel B, in the main text (van Kollenburg and Croon 2022). Unlike Model 2 in Table 4 in the main text, the path model does not comprise the (non-significant) effects of recovery and its interaction with coalition on perceived betrayal.

FIGURE W2
STATISTICAL REPRESENTATION OF THE PATH MODEL FOR TESTING MEDIATED MODERATION

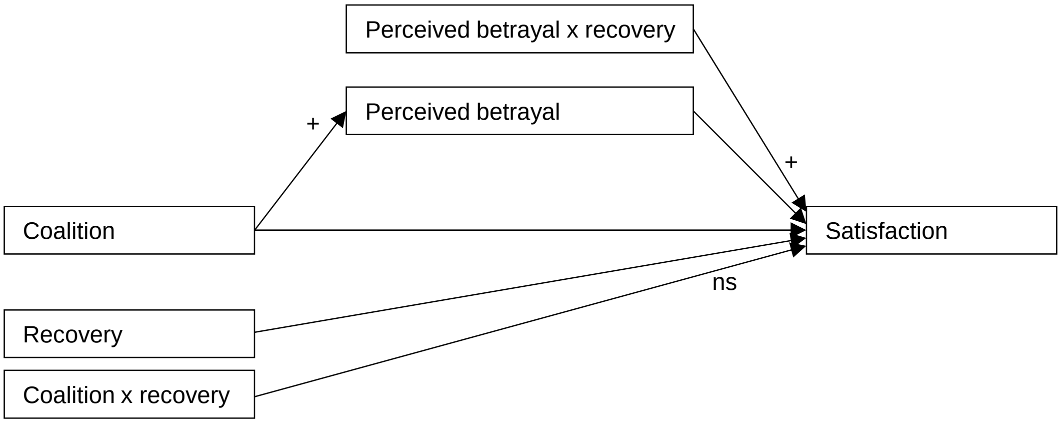

Notes: Coalition: +.5 = TA–SE, −.5 = TA–CO; recovery: +.5 = taking steps, −.5 = apology. Recovery, firm recovery; perceived betrayal, complainers' perceived betrayal by the third actor; satisfaction, complainers' satisfaction with the recovery offered by the firm.

The expected results for the proposed mediated moderation are displayed in Figure W2 via “+” (positive and significant effect) and “ns” (non-significant effect), conditional on the result that the coalition × recovery interaction is significant when tested on its own (van Kollenburg and Croon 2022). For the sake of simplicity, the control variables are not depicted but are included as independent variables of perceived betrayal and satisfaction. To estimate the combined effect of coalition→perceived betrayal and perceived betrayal × recovery→satisfaction, we employed percentile bootstrapping, as recommended by Hayes and Scharkow (2022). The calculations were carried out using IBM SPSS Amos 27.

We ran two path models for Study 2 and Study 3. First, we estimated an unmediated model that assessed the coalition × recovery interaction in the absence of any further effects. Second, we estimated the mediated model, as illustrated in Figure W2. Table W3 presents the results of the path models.

Across Study 2 and Study 3, the results of the unmediated model replicated the coalition × recovery interaction (Study 2: $\beta = .20$, $p = .001$; Study 3: $\beta = .13$, $p = .010$). The mediated models fitted the data satisfactorily (Study 2: $\chi^2[3] = 5.229$, $p = .156$, standardized root mean square residual = .006, root mean square error of approximation = .055; Study 3: $\chi^2[3] = 1.206$, $p = .752$, standardized root mean square residual = .003, root mean square error of approximation < .001; Hu and Bentler 1998). As expected, we found the perceived betrayal × recovery interaction (Study 2: $\beta = .22$, $p = .019$; Study 3: $\beta = .20$, $p = .029$) to be positive and significant, while the coalition × recovery interaction was no longer significant (Study 2: $\beta = .05$, $p = .622$; Study 3: $\beta = -.04$, $p = .655$). In addition, the TA–SE (vs. TA–CO) coalition increased perceived betrayal (Study 2: $\beta = .74$, $p < .001$; Study 3: $\beta = .84$, $p < .001$). Thus, the results obtained using the path analysis approach supported the mediated moderation.

Table W3. Mediated Moderation Results for Study 2 and Study 3, Including all Control Variables.

	Study 2: Tesco				Study 3: Zara			
	Unmediated		Mediated		Unmediated		Mediated	
	Beta	*p*	Beta	*p*	Beta	*p*	Beta	*p*
**Unmediated interaction effects**[a]								
Coalition → satisfaction	.02	.755	-.01	.941	.09	.064	-.01	.898
Recovery → satisfaction	.06	.323	.06	.356	-.36	<.001	-.35	<.001
Coalition × recovery → satisfaction	.20	.001	.05	.622	.13	.010	-.04	.655
**Mediated interaction effects**[a]								
Coalition → perceived betrayal	—	—	.74	<.001	—	—	.84	<.001
Perceived betrayal → satisfaction	—	—	.01	.934	—	—	.13	.188
Perceived betrayal × recovery → satisfaction	—	—	.22	.019	—	—	.20	.029
**Effects of control variables on satisfaction**								
Age	-.07	.330	-.07	.279	.02	.698	.01	.821
Gender (male)[b]	.02	.771	.04	.511	-.10	.069	-.08	.120
Gender (no disclosure)[b]	-.03	.678	-.04	.540	-.03	.495	-.04	.437
Failure magnitude	-.11	.112	-.10	.117	-.07	.189	-.09	.084
Control attributions	-.40	<.001	-.39	<.001	-.23	<.001	-.23	<.001
Attitude toward complaining	.02	.738	.05	.484	.11	.032	.12	.027
Prior experiences[c]	.07	.296	.06	.399	-.07	.214	-.07	.189
Customer contact in daily work	-.09	.161	-.10	.111	-.07	.176	-.07	.132
Self-efficacy	.02	.748	.03	.648	.01	.889	.00	.954
Empathic concern	-.03	.687	-.02	.795	.12	.036	.15	.010
**Effects of control variables on perceived betrayal**								
Age	—	—	.01	.778	—	—	.05	.106
Gender (male)[b]	—	—	.03	.538	—	—	.00	.909
Gender (no disclosure)[b]	—	—	.07	.127	—	—	.01	.619
Failure magnitude	—	—	-.11	.014	—	—	.12	<.001
Control attributions	—	—	.12	.008	—	—	.08	.004
Attitude toward complaining	—	—	-.01	.815	—	—	.01	.832
Prior experiences[c]	—	—	-.07	.121	—	—	.01	.818
Customer contact in daily work	—	—	.01	.854	—	—	.02	.489
Self-efficacy	—	—	-.01	.843	—	—	-.01	.769
Empathic concern	—	—	.05	.368	—	—	-.07	.028
**Model fit**								
χ^2 (df)	—[d]		5.229 (3)		—[d]		1.206 (3)	
p-value	—[d]		.156		—[d]		.752	
SRMR	—[d]		.006		—[d]		.003	
RMSEA	—[d]		.055		—[d]		<.001	

[a]Coalition: +.5 = TA–SE, −.5 = TA–CO; recovery: +.5 = taking steps, −.5 = apology.
[b]Comparing male and "do not wish to disclose" answer options with female as reference category.
[c]Prior experiences with Facebook and fashion stores for Studies 2 and 3, respectively.
[d]Not available because the model is saturated (df = 0).
Notes: Recovery, firm recovery; perceived betrayal, complainers' perceived betrayal by the third actor; satisfaction, complainers' satisfaction with the recovery by the firm.

Additional References for Theme 4 of the Web Appendix

van Kollenburg, Geert H. and Marcel A. Croon (2022), "How to Define and Test an Indirect Moderation Model: The Missing Link in Regression-Based Path Models," *Methodology*, 18 (3), 167–84.

Hayes, Andrew F. and Michael Scharkow (2013), "The Relative Trustworthiness of Inferential Tests of the Indirect Effect in Statistical Mediation Analysis: Does Method Really Matter?" *Psychological Science*, 24 (10), 1918–27.

Hu, Li-tze and Peter M. Bentler (1998), "Fit Indices in Covariance Structure Modeling: Sensitivity to Underparameterized Model Misspecification," *Psychological Methods*, 3 (4), 424–53.
