# Supplementary material for: Coalitions and Their Negative Consequences: An Examination in Service Failure-Recovery Situations
Source: J Serv Res. 2023 Mar 30;26(4):614–35. doi: 10.1177/10946705231163884 (PMC10522451; doi:10.1177/10946705231163884)
Supplement: Supplemental Material - Coalitions and Their Negative Consequences: An Examination in Service Failure-Recovery Situations [file sj-pdf-2-jsr-10.1177_10946705231163884.pdf]

What Happens When Customers Take Side With the Complainer or the Firm During Service Failure Interactions?

Using a large field-study and two experiments, Holger Roschk, Masoumeh Hosseinpour and Jan Breitsohl examine the consequences of customers who form coalitions by taking sides with a complainer or a firm during service failure situations. Key insights: Coalitions are always harmful, but there are limits to, and remedies for, the negative impact.

The increasingly social nature of customer experiences, such as when complaining on social media, creates ample opportunities for other customers to side with the complainer or the firm—that is, to form a coalition. The present research is the first to examine how such coalitions affect service failure and recovery dynamics, and is published today in the highly regarded Journal of Service Research. The study conceptualized other customers as a third actor and examines two conceivable coalitions: between the third actor and complainer and between the third actor and service employee.

In their study, Holger Roschk (Professor at Aalborg University), Masoumeh Hosseinpour (Assistant Professor at Aarhus University), and Jan Breitsohl (Associate Professor at University of Glasgow) demonstrate that coalitions are a pervasive phenomenon in service failure settings. The authors analyzed the official Facebook pages of 17 retailers over a period of four months. Results show that coalitions occurred in 32% (±3.5%) of complaint episodes. The formation of a coalition after a complaint leads to a downward trajectory of 16%–32% in the affective tone of an online conversation, shifting the tone from neutral to negative. The occurrence of coalitions also impairs firms' recovery efforts and shifts complainers' recovery preferences.

Nevertheless, the authors also found evidence that the negative impact of coalitions can be mitigated. While both third actor–complainer and third actor–service employee coalitions deteriorate the affective tone of an online conversation, the joint impact of the two coalitions is not more harmful than that of each coalition on its own. In other words, there is a lower limit in the coalition-induced downward trajectory. Cautiously, the results also indicate that the firm responses might mitigate some of the detrimental impact of subsequently present coalitions.

Leveraging their findings, the authors recommend an adaptive recovery approach which may partially mitigate the negative impact of coalitions. Specifically for online conversations, the authors suggest firms to offer a speedy response that conveys accommodative content in a personal and positive manner. Coalitions change complainers' recovery preferences, and firms are recommended to emphasize the steps they took or will take to solve the problem in situations where others side with the service employee, and to offer an apology alongside an explanation of what happened when others side with the complainer.

Overall, the authors conclude that coalitions are a salient and detrimental social dynamic in failure situations to which firms can and need to adapt. These findings are a first step in studying coalitions, and the authors recommend a set of further exciting questions that are worth investigating for both service practitioners and scholars.
